# Precursor central memory versus effector cell fate and naïve CD4^+^ T cell heterogeneity

**DOI:** 10.1084/jem.20231193

**Published:** 2024-09-25

**Authors:** Deeksha Deep, Herman Gudjonson, Chrysothemis C. Brown, Samuel A. Rose, Roshan Sharma, Yoselin A. Paucar Iza, Seunghee Hong, Saskia Hemmers, Michail Schizas, Zhong-Min Wang, Yuezhou Chen, Duane R. Wesemann, Virginia Pascual, Dana Pe’er, Alexander Y. Rudensky

**Affiliations:** 1 Immunology Program, Memorial Sloan Kettering Cancer Center, New York, NY, USA; 2 Tri-Institutional MD-PhD Program, Weill Cornell Medicine, The Rockefeller University and Memorial Sloan Kettering Cancer Center, New York, NY, USA; 3 Immunology and Microbial Pathogenesis Program, Weill Cornell Medicine Graduate School of Medical Sciences, New York, NY, USA; 4 Computational and Systems Biology Program, Memorial Sloan Kettering Cancer Center, New York, NY, USA; 5 Immuno-Oncology, Human Oncology and Pathogenesis Program, Memorial Sloan Kettering Cancer Center, New York, NY, USA; 6 Department of Pediatrics, Memorial Sloan Kettering Cancer Center, New York, NY, USA; 7 Drukier Institute for Children’s Health at Weill Cornell Medicine, New York, NY, USA; 8 Department of Integrative Immunobiology, Duke University School of Medicine, Durham, NC, USA; 9 Gerstner Sloan Kettering Graduate School in Biomedical Sciences, Memorial Sloan Kettering Cancer Center, New York, NY, USA; 10 Immunology Discovery, Genentech Inc., South San Francisco, CA, USA; 11 Department of Medicine, Division of Allergy and Immunology, Division of Genetics, Brigham and Women’s Hospital, Harvard Medical School, Boston, MA, USA; 12 Howard Hughes Medical Institute, Chevy Chase, MD, USA

## Abstract

Upon antigenic stimulation, naïve CD4^+^ T cells can give rise to phenotypically distinct effector T helper cells and long-lived memory T cells. We computationally reconstructed the in vivo trajectory of CD4^+^ T cell differentiation during a type I inflammatory immune response and identified two distinct differentiation paths for effector and precursor central memory T cells arising directly from naïve CD4^+^ T cells. Unexpectedly, our studies revealed heterogeneity among naïve CD4^+^ T cells, which are typically considered homogeneous save for their diverse T cell receptor usage. Specifically, a previously unappreciated population of naïve CD4^+^ T cells sensing environmental type I IFN exhibited distinct activation thresholds, suggesting that naïve CD4^+^ T cell differentiation potential may be influenced by environmental cues. This population was expanded in human viral infection and type I IFN response-lined autoimmunity. Understanding the relevance of naïve T cell heterogeneity to beneficial and maladaptive T cell responses may have therapeutic implications for adoptive T cell therapies in cancer immunotherapy and vaccination.

## Teaser

Trajectory analysis of CD4^*+*^ T cell differentiation during a type I inflammatory immune response identified two distinct differentiation paths for effector and precursor central memory T cells arising from heterogeneous naïve CD4^*+*^ T cells.

## Introduction

CD4^+^ T cells, principal regulators of both the magnitude and type of immune response, emerge from the thymus as quiescent naïve cells. Upon infectious challenge, naïve CD4^+^ T cells give rise to an array of relatively short-lived effector and long-lived memory cells. Two models have been proposed for the emergence of central memory T (T_CM_) cells: a linear model in which naïve T cells, upon activation, first differentiate into effector cells, a subset of which transitions to memory cells; or a branching model, whereby activated naïve T cells generate both effector and memory T cell progeny during their initial cell divisions. While recent studies characterizing CD4^+^ T cell heterogeneity during acute viral infection identified the presence of precursor T_CM_ (pT_CM_) at a single time point of the analysis, such a snapshot approach precluded assessment of their developmental dynamics. Thus, the current view of pT_CM_ fate decisions remains ambiguous.

Although the cues that regulate CD4^+^ T cell effector lineage commitment have been extensively studied over the last two decades, the signals that influence the differentiation of memory CD4^+^ T cells either from naïve or effector cells are poorly understood. Numerous studies have suggested that T cell receptor (TCR) affinity serves as the key determinant of T cell fate with increased TCR signal strength associated with T_H_1 versus T follicular helper (T_FH_) or pT_CM_ differentiation (Tubo et al., 2013). This notion was recently called into question by a study utilizing single-cell TCR sequencing (scTCR-seq) to track the fate of individual virus-specific T cells in which the majority of naïve T cell clonotypes were identified within both T_H_1 and T_FH_ effector cell populations at the peak of the responses, yet at the same time a sizeable proportion of virus-specific clones displayed T_H_1 or T_FH_ restriction (Khatun et al., 2021).

To explore the temporal and developmental relationships between naïve, effector, and memory T cells, and the role of environmental cues versus TCR specificity in driving these distinct cell fates, we deployed paired single-cell RNA and TCR sequencing (scRNA/scTCR-seq) combined with trajectory analyses to reconstruct naïve CD4^+^ T cell differentiation during acute bacterial infection (Nowotschin et al., 2019). Our analysis defined the differentiation trajectories and associated signaling pathways from naïve to effector T cell lineages and precursor central memory cells, revealing an early branch of pT_CM_ cells arising directly from naïve T cells. These lineage fate decisions were not determined by the TCR specificities of naïve CD4 T cells. Interrogation of the signaling pathways associated with pT_CM_ versus effector T cell differentiation revealed that sustained type I IFN signaling following priming of naïve CD4^+^ T cells was associated with the generation of precursor memory CD4^+^ T cells, suggesting that divergent effector and memory T cell fates may originate from distinct cellular niches. Surprisingly, our analyses also uncovered pre-existing heterogeneity within the naïve CD4^+^ T cell compartment in mice: notably a population of naïve cells experiencing type I IFN signaling as well as previously described CD5^hi^ naïve T cells (Weber et al., 2012; Persaud et al., 2014; Mandl et al., 2013; Bartleson et al., 2020), each poised for distinct fates upon activation. Examination of human T cells either during acute viral infection or systemic lupus erythematosus (SLE), a type I IFN–associated autoimmune disease, suggested dynamic responsiveness of naïve T cells to type I IFN and likely other environmental cues. We posit that the naïve T cell pool represents a mix of cells with differing “life histories,” reflecting their exposure to infectious and other environmental perturbations potentially experienced as bystanders during their residence in, or passage through, anatomically distinct LNs with unique cellular and lymph composition providing a rich source of factors with immune-modulatory potential (Esterházy et al., 2019).

## Results

### Heterogeneity of effector CD4^+^ T cell states and relationship to precursor central memory T cells

**Figure 1.**
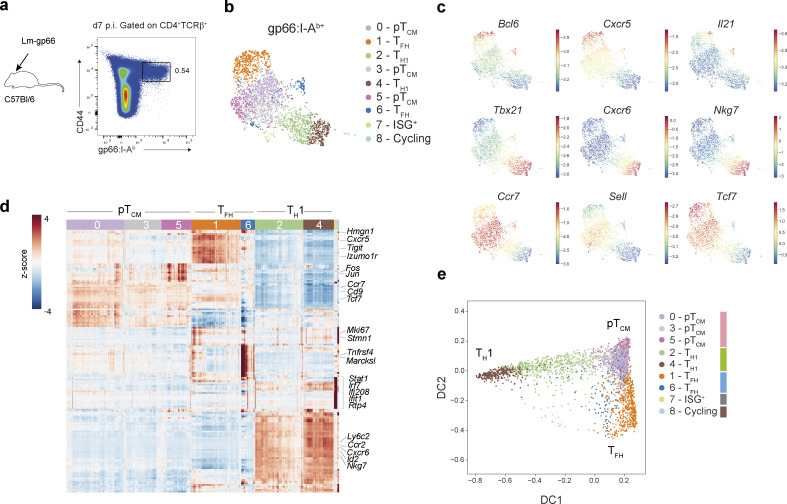
**Effector memory CD4**^**+**^ **T cell heterogeneity during acute bacterial infection. (a)** Strategy to isolate wild-type gp66:I-A^b+^ CD4^+^ T cells for scRNA-seq, 7 days after infection with L.m.-gp66. **(b)** UMAP of 2964 gp66:I-A^b^-specific CD4^+^ T cells colored by Phenograph clusters and annotated by inferred CD4^+^ T cell effector/memory lineage. **(c)** UMAP overlaid with imputed expression of canonical T_FH_, T_H_1, or T_CM_ genes. **(d)** Imputed, log-normalized expression of top 20 DEGs (log_2_FC > 0.5, FDR < 0.01) for each phenograph cluster shown in Fig. 1 b. Colored bar at the top of the heatmap indicates cluster assignments. **(e)** Diffusion map of gp66:I-A^b^-specific CD4^+^ T cells using the first two DCs reflecting distinct CD4^+^ T cell fates. Cells are colored by cluster as in Fig. 1 b.

During infection with a type 1 immune response-inducing pathogen, activation of naïve pathogen-specific CD4^+^ T cells results in their differentiation into two major effector cell states: T_H_1 cells and T_FH_ cells. In addition, a subset of T_CM_ cells also emerges (Pepper et al., 2011). Previous studies have proposed that the choice between particular flavors of effector and memory T cell responses is determined by the strength of the TCR signal (Tubo et al., 2013). To determine the full extent of effector T cell heterogeneity during acute bacterial infection and resolve contributions of TCR usage-based cell intrinsic versus extrinsic cues in directing naïve T cell fates, we infected B6 mice with *Listeria monocytogenes* (L.m.) expressing lymphocytic choriomeningitis virus (LCMV) envelope glycoprotein-derived antigenic peptide gp66-80 (L.m.-gp66) and performed scRNA-seq and TCR-seq on FACS purified I-A^b^:gp66 tetramer-bound effector CD4^+^ T cells on day 7 after infection (Fig. 1 a).

**Figure S1.**
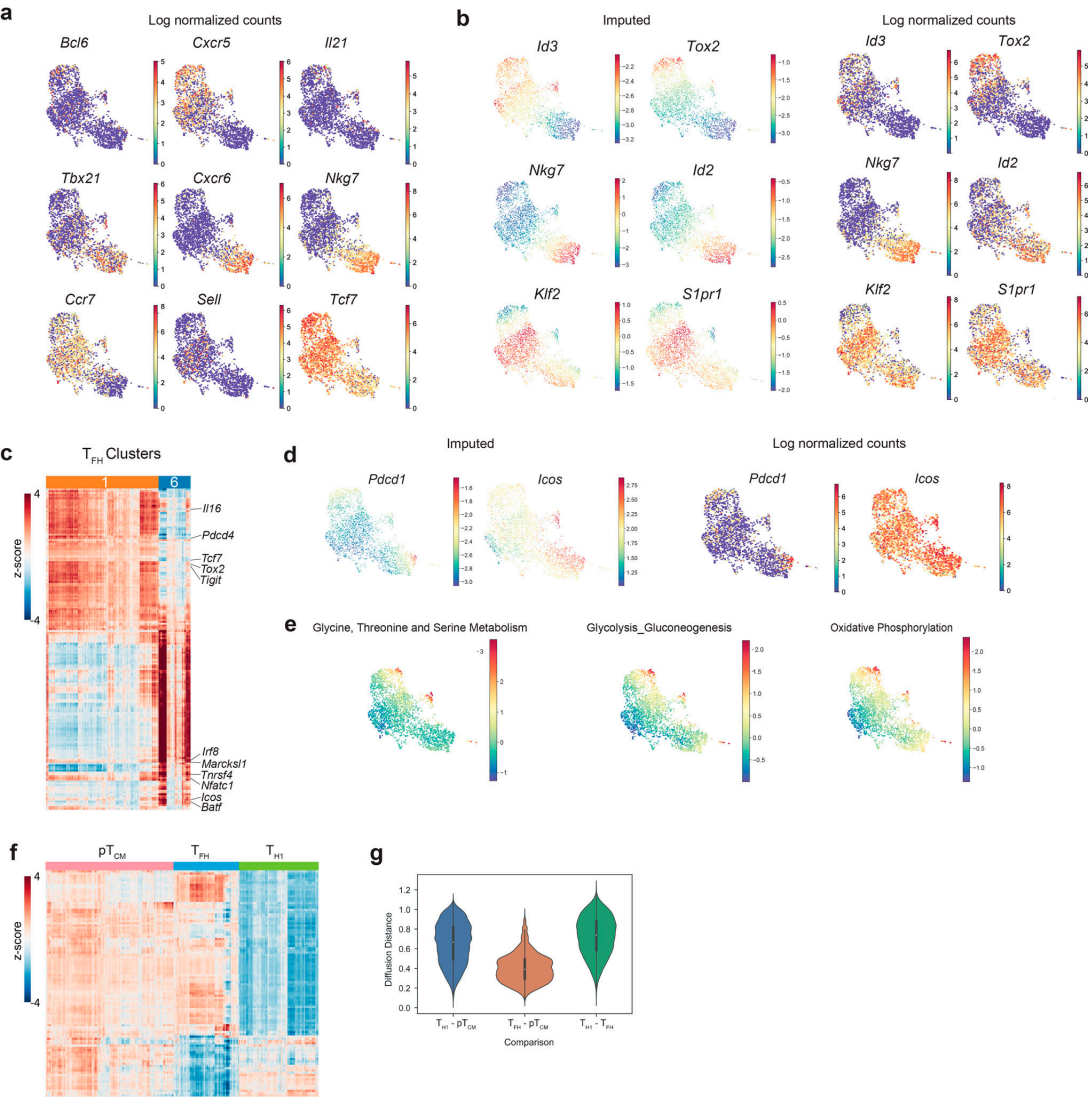
**Analysis of CD4**^**+**^** T cell responses to systemic L.m. infection.** Related to Fig. 1.** (a)** UMAP overlaid with log normalized counts of canonical T_FH_, T_H_1, or T_CM_ genes for comparison with imputed expression Fig. 1 c. **(b)** UMAP overlaid with imputed (left) and log normalized (right) expression of T_FH_, T_H_1, or T_CM_ genes. **(c)** Heatmap showing imputed, log-normalized expression of all DEGs (log_2_FC > 0.5, FDR < 0.01) identified for each T_FH_ cluster (cluster 6 or cluster 1). The 177 differentially expressed T_FH_ genes shown include the union of DEGs identified in each replicate in one vs. rest comparisons for each T_FH_ cluster. The colored bar at the top of the heatmap shows the assignment of cells to these clusters. **(d)** UMAP of single-cell transcriptomes from gp66:I-A^b+^ CD4^+^ T cells colored by imputed (left) and log-normalized (right) expression of canonical germinal center T_FH_ genes. **(e)** UMAP overlaid with the mean expression of genes from the indicated KEGG pathway geneset. **(f)** Heatmap showing pT_CM_-specific gene expression. Heatmap shows imputed, log-normalized expression of all DEGs (log_2_FC > 0.5, FDR < 0.01) in pT_CM_ versus T_FH_ or pT_CM_ versus T_H_1 comparisons. pT_CM_, T_FH_, and T_H_1 clusters were merged prior to differential gene expression calculation. **(g)** Diffusion distances between pair-wise comparisons of T_FH_, T_H_1, and T_CM_ cells.

Analysis of scRNA-seq profiles and TCR usage of effector T cells (2,964 and 2,465 cells, respectively) identified nine clusters (Fig. 1 b). Based on the differential expression of canonical T cell lineage genes, three distinct T cell types we identified included T_H_1, T_FH_, and a third cell type, expressing T_CM_ markers CCR7 and CD62L (Fig. 1, c and d; and Fig. S1 a). pT_CM_ cells were further distinguished by increased expression of *Klf2* and *S1pr1* genes (Fig. S1 b), a profile consistent with recirculating T_CM_ (Skon et al., 2013). Visualization of T cell phenotypes using diffusion maps demonstrated a phenotypic continuum between pT_CM_ cells and either T_H_1 or T_FH_ cells (Fig. 1 e).

Differential gene expression analysis for T_FH_, T_H_1, and pT_CM_ cells identified T_H_1 lineage-specific genes, including exclusive expression of *Tbx21*, *Ly6c2*, *Nkg7*, and *Id2*, as well as the chemokine receptor gene *Cxcr6* while T_FH_ cells expressed *Id3*, *Bcl6*, and *Cxcr5* (Fig. 1, c and d; and Fig. S1, a and b). These phenotypes are consistent with those identified in a study that also analyzed the spectrum of I-A^b^:gp66-specific polyclonal effector CD4^+^ T cells in acute viral infection with LCMV Armstrong and Clone 13 (Andreatta et al., 2022). CXCR5^+^Bcl6^+^ T_FH_ cells encompassed two transcriptionally distinct clusters: 1 and 6. T_FH_ cluster 1 was distinguished by increased expression of canonical T_FH_ genes (*Tcf7*, *Tox2*, *Id3*, *Izumo1r*) and genes encoding immune inhibitory receptors, *Tigit* and *Cd200,* whereas T_FH_ cluster 6 exhibited increased expression of TCR-dependent genes *Tnfrsf4* (OX40), *Batf*, *Irf8*, as well as *Marcksl*, a critical regulator of cell migration (Fig. S1 c). At this time point, we did not observe evidence of *Pdcd1* expression, although cells in cluster 6 expressed *Icos* (Fig. S1 d). In addition, cluster 6 cells were enriched for the expression of genes related to amino acid metabolism, and glycolytic and oxidative phosphorylation pathways (Fig. S1 e), suggesting that these metabolically active cells represent pre-germinal center T_FH_ cells (Merkenschlager et al., 2021). Interestingly, pT_CM_ cells also expressed signature T_FH_ genes including *Tcf7* and *Id3* (Fig. 1 c and Fig. S1 b). Surprisingly, Id3 expression was also recently found to be a relevant marker of memory potential amongst T_H_1 cells (Shaw et al., 2022). Our differential gene expression analysis revealed remarkable transcriptional overlap between pT_CM_ and T_FH_ cells, with very few gene expression features distinguishing these two subsets (Fig. S1 f). Furthermore, analysis of diffusion distances, a measure of phenotypic similarity, demonstrated increased proximity between T_FH_ and pT_CM_ cells compared with T_FH_ and T_H_1 cells or T_H_1 and pT_CM_ cells (Fig. S1 g), suggesting a closer developmental relationship between pT_CM_ and T_FH_ versus pT_CM_ and T_H_1 cells.

### Multipotentiality of TCR clonotypes

**Figure 2.**
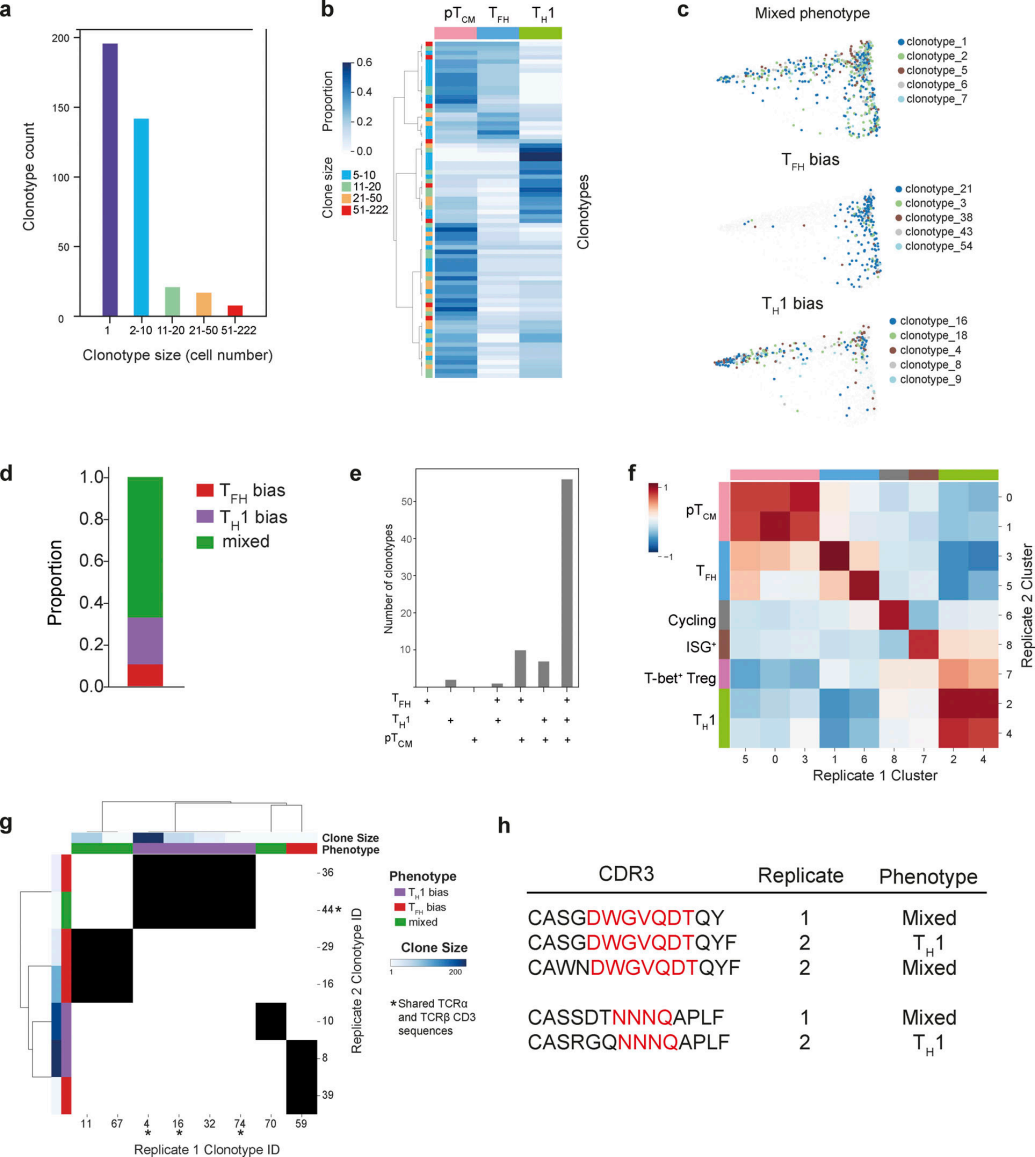
**CD4**^**+**^ **T cell fate is independent of TCR specificity. (a)** Frequency and sizes of clonotypes amongst gp66:I-A^b^-specific CD4^+^ T cells. **(b)** Proportion of cells with a T_H_1, T_FH_, or pT_CM_ phenotype for each expanded clonotype (≥5 cells). Each row represents an individual clonotype. Clonotypes are ordered by hierarchical clustering with complete linkage and correlation distance. **(c)** Diffusion map of gp66:I-A^b+^ CD4^+^ T cells overlaid with the five largest clonotypes for each clonotype-phenotype pattern. **(d)** Proportion of clonotypes (≥5 cells) exhibiting bias toward a particular T_H_ cell lineage. Clonotypes exhibiting no lineage bias are labeled as “mixed.” **(e)** Observed number of clonotypes with cells distributed across the T_FH_, T_H_1, and T_CM_ phenotypes indicated along the x axis. **(f)** Shared gene signatures representing T_H_ lineages across two independent experiments identifying matched clusters. Pearson correlation between transcriptomes of replicate gp66:I-A^b^-specific CD4^+^ T cell clusters demonstrating high concordance between independent samples. **(g)** Shared clonotypes with distinct phenotypes in biological replicate samples. Each depicted clonotype has ≥5 cells and an overlap in TCRα, TCRβ, or paired TCRα/TCRβ sequences across replicate samples. Matching clonotypes between replicate samples indicated by black shading. Shared paired TCRα and TCRβ CDR3 sequences, but distinct phenotypes across replicate samples (clonotype 44 [replicate 2], and clonotypes 4, 74, and 16 [replicate 1]) are indicated by an asterisk. **(h)** CDR3 sequences and T_H_ lineage bias for clonotypes with a shared TCR specificity group across the two biological replicate samples.

**Figure S2.**
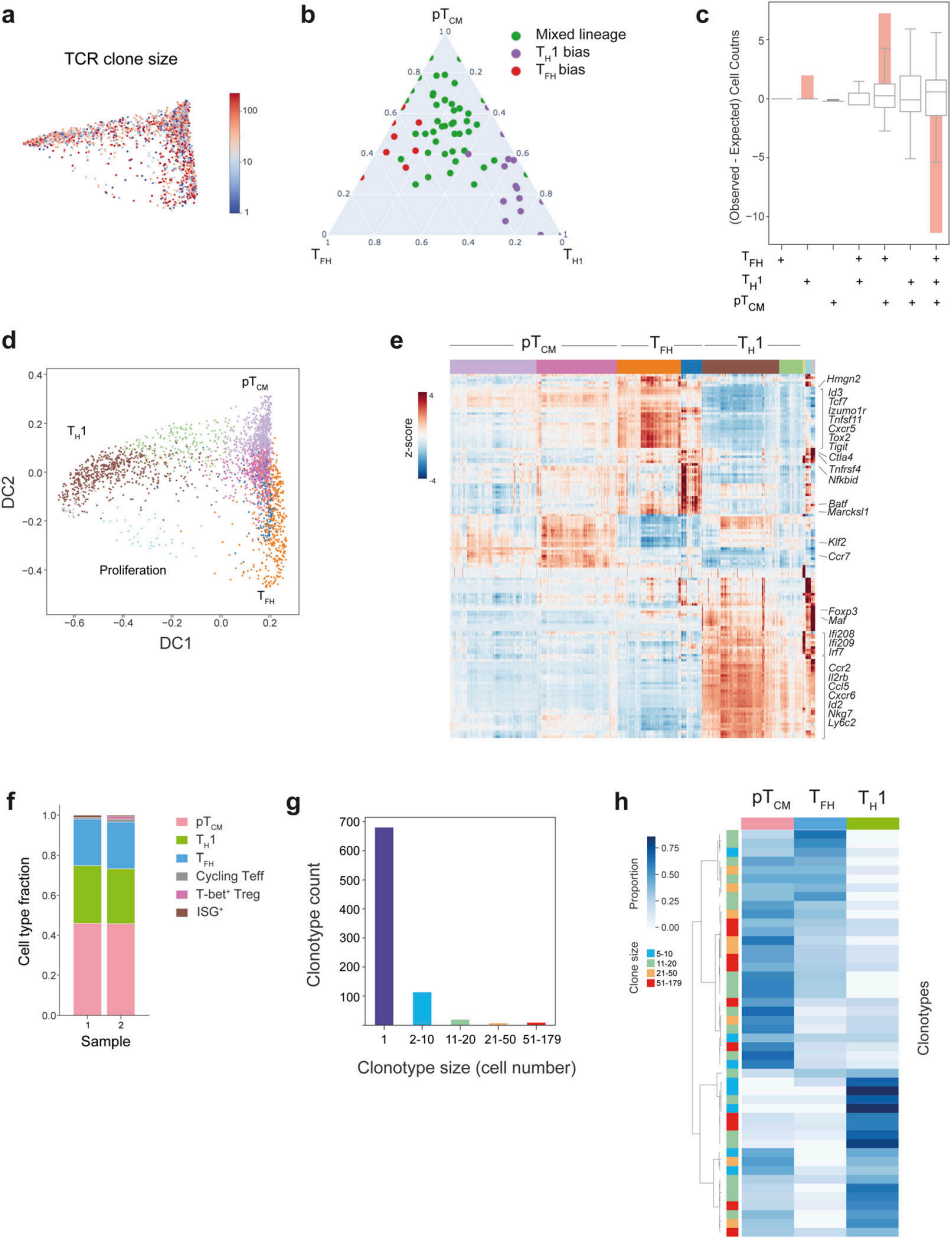
**Paired TCR and transcriptomic analysis of antigen-specific CD4**^**+**^** T cell responses to L.m.** Related to Fig. 2. **(a)** Diffusion map of gp66:I-A^b+^ CD4^+^ T cells colored by clone size, showing a similar degree of clonal expansion across T_H_ cell subsets. **(b)** Clonotypes ≥5 cells were clustered on the basis of their phenotypic distribution using Phenograph. Three distinct phenotypic patterns were identified: T_H_1 bias, T_FH_ bias, or mixed lineage phenotypes. Ternary plot showing the proportion of T_H_1, T_FH_, or pT_CM_ cells for each clonotype. Each dot represents an individual clonotype, colored by its phenotype. **(c)** Bar graph showing the overrepresentation or underrepresentation (observed–expected counts) of clonotype frequency for each combination of T_FH_, T_H_1, and T_CM_ phenotypes with respect to randomized permutations. Box plots indicate the expected clonotype frequencies for each phenotype combination if clones were randomly distributed. The solid pink bars represent the deviation of the observed clonotype frequency for each phenotype combination from the randomized expectation. **(d)** Diffusion map visualization of single cell transcriptomes from replicate gp66:I-A^b+^ CD4^+^ T cells, colored by their Phenograph cluster identity, illustrating three distinct cell fates: T_H_1, T_FH_, and pT_CM_. **(e)** Heatmap showing MAGIC imputed, log-normalized expression of top 20 DEGs (log_2_FC > 0.5, FDR < 0.01), for each Phenograph cluster shown in d. The colored bar at the top of the heatmap shows the assignment of cells to these clusters. **(f)** Fraction of cells within each effector CD4^+^ T cell lineage for replicate samples. Equivalent proportions of T_H_1, T_FH_, and pT_CM_ cells were observed in two independent experiments. **(g)** Graph showing frequency and size distribution of clonotypes amongst replicate gp66:I-A^b+^ CD4^+^ T cells. **(h)** Heatmap demonstrating the proportion of cells within a given clonotype with a T_H_1, T_FH_, or pT_CM_ phenotype, for clonotypes ≥5 cells for the replicate gp66:I-A^b+^ CD4^+^ T cell sample. Each row represents an individual clonotype. The color bar on the left indicates clone size. Clonotypes are ordered by hierarchical clustering with complete linkage and correlation distance.

To determine the role of TCR specificity in T cell fate and the developmental relationship between pT_CM_ and effector populations, we tracked the fate of individual naïve T cells by analyzing their TCR utilization. Amongst CD4^+^ T cells recognizing a specific peptide-MHC (gp-66:I-A^b^), we identified 384 unique clones, 188 of which were represented by two or more cells (Fig. 2 a and Fig. S2 a). Cells with the same TCR were present in multiple clusters spanning T_H_1, T_FH_, and pT_CM_ phenotypes (Fig. 2 b). Clustering clonotypes ≥5 cells on the basis of phenotypic distribution to look for lineage overlap revealed three distinct archetypes (Fig. 2, c and d; and Fig. S2 b). The majority of clonotypes exhibited no lineage bias, indicating that a single naïve CD4^+^ T cell can differentiate into multiple effector states. However, a proportion of clonotypes were predominantly associated with a T_H_1 or T_FH_ phenotype. Given that CD4^+^ T cell fate determination is temporally associated with cell division, it is perhaps not surprising that lineage-committed cells will produce progeny of the same type.

The lineage relationship between effector and memory CD4^+^ T cells has been a matter of debate. A branching model whereby memory and effector T cells arise from a single naïve T cell during the first cell division, and a linear model, whereby effector cells give rise to memory or vice versa, have both been proposed. To resolve this relationship, we analyzed clonal overlap across combinations of effector and memory subsets (Fig. 2 e). Although a small proportion of clones represented CD4^+^ T cells that had exclusively adopted a T_H_1 fate, no clones were exclusive to T_FH_ or pT_CM_ lineages (Fig. S2 c). Amongst clonotypes that were only present in pairwise combinations of cells, we observed a significant enrichment of clones composed of both T_FH_ and pT_CM_ cells (Fig. 2 e). Furthermore, amongst clonotypes only present in two cell types, the combination of T_FH_ and pT_CM_ cells was significantly enriched, further indicating a close developmental relationship between these two states (Fig. 2 e).

We reasoned that if TCR specificity influences T cell fate, cells with identical TCR clonotypes across multiple experiments would also share transcriptional phenotypes. We therefore performed combined scRNA/TCR-seq analyses on an independent replicate of 2,717 cells with paired TCR sequences for 2,663 cells. We identified nine clusters (Fig. S2, d and e) with essentially identical gene expression signatures to the clusters in the first experiment (Fig. 2 f) and equivalent proportions of T_H_1, T_FH_, and pT_CM_ cells (Fig. S2 f). The replicate sample contained 833 unique clones (152 represented by two or more cells), which were distributed across distinct phenotypes (Fig. S2, g and h), and 28 clonotypes shared TCRα, TCRβ, or paired TCRα/TCRβ nucleotide sequences across the two samples. Notably, amongst shared clones with ≥5 cells, we observed divergent lineage bias between the two independent experiments with clones associating with distinct cellular phenotypes (Fig. 2 g). We extended this analysis using the GLIPH (grouping of lymphocyte interactions by paratope hotspots) tool to identify cells with putative shared antigen specificity based on shared motifs within CDR3 (Glanville et al., 2017). This identified cells with an additional specificity group, shared across the two experiments, which also exhibited distinct phenotypic patterns between replicates (Fig. 2 h). Thus, these results show that CD4^+^ T cell clones with the same specificity independently arising in individual animals may adopt different fates, which is consistent with several recent studies demonstrating that even though some TCR clonotypes may display a lineage preference, the majority do not (Khatun et al., 2021; Andreatta et al., 2022).

Although the variables related to TCR signaling strength, including ligand density, co-stimulatory molecules, and cytokine signaling, may impact predisposition to memory versus effector T cell differentiation, our experiments demonstrate that TCR specificity is not a primary determinant of fate. While some TCR clonotypes did exhibit lineage preference, many were multipotential across two independent experiments, indicating microenvironmental signals received before or during T cell activation play a predominant role in effector T cell lineage choice.

### A pT_CM_ differentiation pathway arising from naïve CD4^+^ T cells

**Figure 3.**
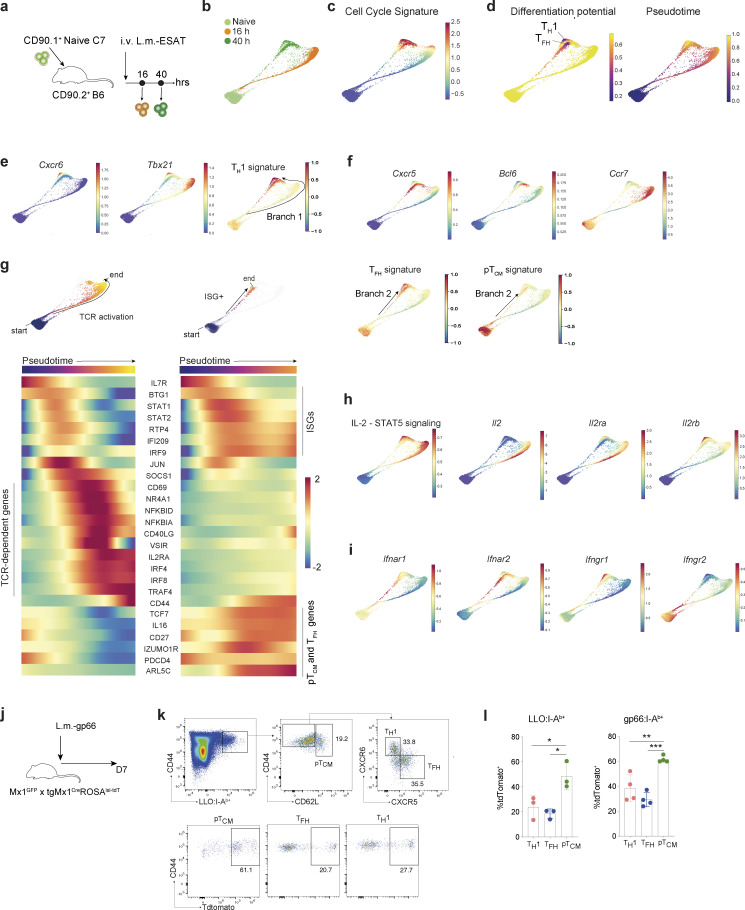
**Emergence of pT**_**CM**_ **from naïve CD4**^**+**^ **T cells. (a)** Experimental strategy to study in vivo naïve C7 TCR transgenic CD4^+^ T cell differentiation during acute L.m.*-*ESAT infection. **(b–i)** Force-directed layout, following Harmony normalization, of naïve and effector C7 CD4^+^ T cells, sampled 16 and 40 h after infection with L.m.-ESAT, and overlaid by different coloring schemes: **(b)** time of sampling. **(c)** Expression of a cell cycle (G1/S and G2/M) gene signature (d) Palantir differentiation potential (left panel) and pseudotime (right panel) using a quiescent (*Ccr7*^hi^*Il7r*^hi^) naïve start cell, demonstrating two regions of reduced differentiation potential that indicate lineage specification and commitment. **(e)** Expression of genes associated with T_H_1 CD4^+^ T cell lineage and average expression of T_H_1 signature genes with delineation of a proposed Branch 1. **(f)** Expression of genes associated with T_FH_ or pT_CM_ CD4^+^ T cell lineages and average expression of T_FH_ or pT_CM_ signature genes with delineation of a proposed Branch 2 (g) Pseudotemporal ordering of alternative differentiation trajectories for naïve CD4^+^ T cells, showing selected start and end points on the force directed layouts (top panels). Heatmaps depict inferred temporal gene expression trends along the two differentiation pathways; left represents cells with high TCR signaling-dependent gene expression (“TCR-hi”), right represents “TCR-lo” pT_CM_ differentiation. **(h)** Force-directed layouts colored by expression of genes associated with IL-2 -STAT5 signaling. **(i)** Type I IFN and IFN-γ receptor gene expression, overlaid on CD4^+^ T cell force-directed layout. **(j)** C7 × *Mx1*^*GFP*^ × tg*Mx1*^*Cre*^*Rosa26*^*lsl-tdT*^ mice were infected with L.m.-gp66 and analyzed 7 days post infection (dpi). **(k)** Representative flow cytometric analysis of Listeriolysin O (LLO) peptide-specific CD4^+^ T cells demonstrating increased proportion of *Mx1* fate-mapped cells amongst CD62L^+^ pT_CM_ cells. **(l)** Proportion of LLO:I-A^b^- (left) and gp66:I-A^b^-specific effector memory CD4^+^ T cell subsets that are *Mx1* fate-mapped. Each symbol represents an individual mouse (l). Data from one of two experiments (l) Error bars: means ± SEM of replicates. Statistical significance determined by one-way ANOVA (l); *P < 0.05; **P < 0.01; ***P < 0.001.

**Figure S3.**
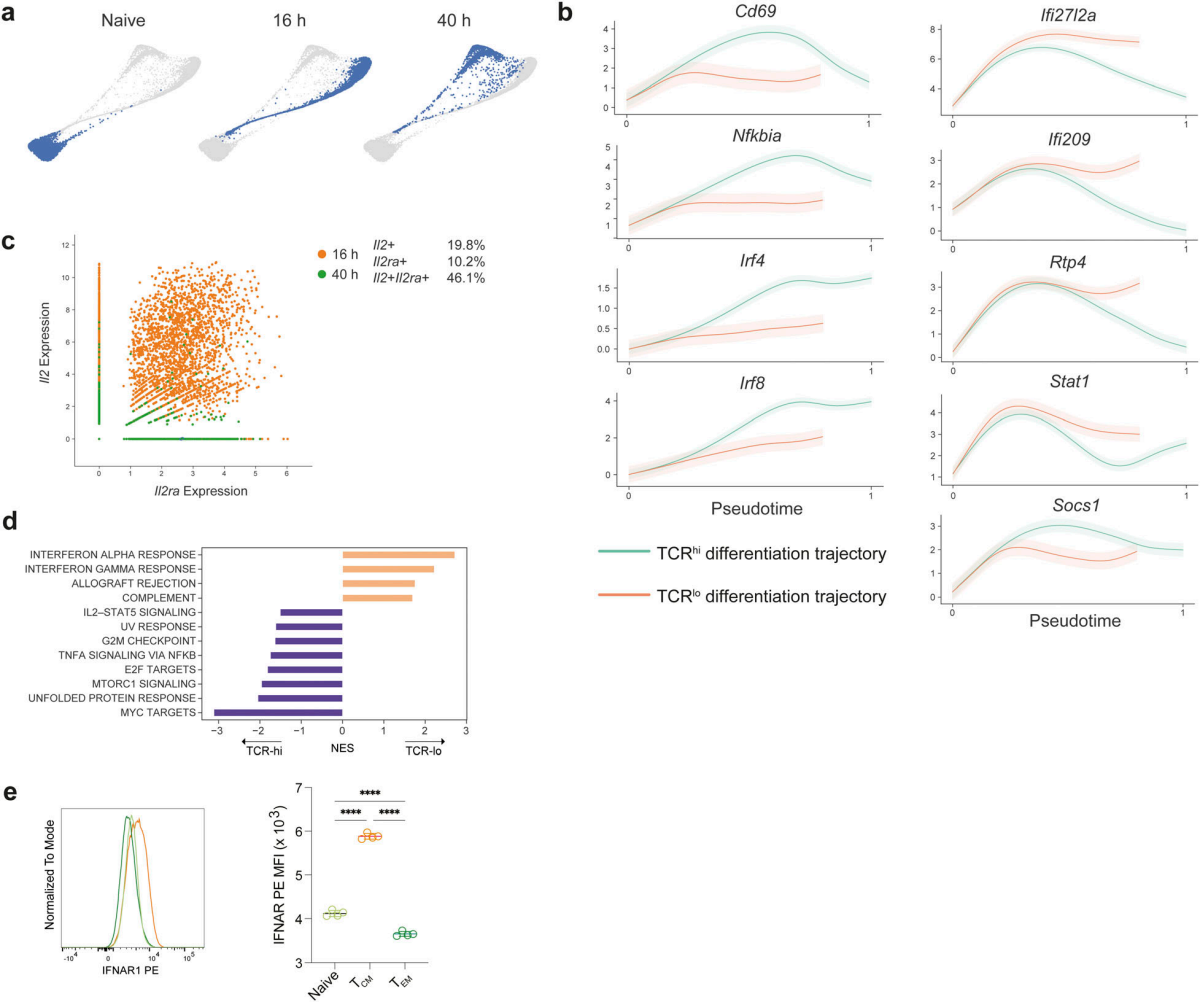
**Pseudotemporal analysis of CD4^+^ T cell fates during L.m. infection.** Related to Fig. 3. **(a)** Force-directed layout depicting the developmental relationship between C7 CD4^+^ naïve and effector T cells during infection with L.m.-ESAT. Cells are colored by sampling time point after infection. **(b)** Gene expression trends along the two branches of T effector/memory differentiation. Cells exhibiting low levels of TCR-dependent genes (“TCR-lo”) exhibit sustained expression of ISGs. **(c)** Unimputed log normalized expression of *Il2* and *Il2ra* for individual C7 effectors profiled at 16 and 40 h post activation, demonstrating coexpression of these two genes. Each dot represents an individual cell colored by the time-point of sampling. Percentages of cells expressing *Il2*, *Il2ra*, or both are listed on the right. **(d)** Enrichment of MsigDB Hallmark pathways in “TCR-lo” (pT_CM_) versus “TCR-hi” (effector) differentiation branches. **(e)** Histogram of IFNAR1 staining of naïve CD4^+^ T cells, T_CM_ and T_EM_ (left) and quantification (right). Representative of two independent experiments. Statistical significance determined by one-way ANOVA; ****P < 0.0001.

To investigate the environmental signals directing CD4^+^ T cell heterogeneity, we adoptively transferred naïve CD90.1^+^ C7 T cells into B6 hosts and either parked them in the new host without challenge for 7 days or subjected them to antigenic challenge the next day upon infection with an attenuated strain of *L*.* monocytogenes* engineered to secrete the mycobacterial protein ESAT-6 (L.m.-ESAT) containing the cognate antigen for the C7 TCR (Fig. 3 a) (Gallegos et al., 2008). We used the Harmony tool (Nowotschin et al., 2019) to stitch together scRNA-seq time points from naïve C7 T cells and C7 T cells at 16 and 40 h post-infection (hpi) and reconstructed continuous “naïve-to-effector” differentiation trajectories (Fig. 3 b and Fig. S3 a). Proliferation, as determined by enrichment of cell-cycle genes, was first evident at 16 hpi, with ∼65% of cells proliferating at 40 hpi (Fig. 3 c).

To characterize gene expression and fate dynamics along the trajectories, we combined Harmony and Palantir (Setty et al., 2019; Nowotschin et al., 2019) analyses specifying a quiescent naïve cell as the “start cell state.” This approach revealed two terminal cell states (Fig. 3 d), distinguished by T_H_1 or T_FH_ signature genes (Fig. 3, e and f), demonstrating lineage divergence within 40 h of infection. Intriguingly, we identified two differentiation pathways that bifurcate from an “isthmus” connecting cells recently downregulating naïve T cell markers (*Il7r*) to early effector/memory precursors. Remarkably, these two branches were composed of cells present at different time points after infection. Branch 1 consisted of cells present at 16 hpi, which expressed genes related to T_H_1 cells and progressed toward the T_H_1 terminal state by 40 hpi (Fig. 3, b and e); branch 2 was composed only of cells present at 40 hpi, which expressed genes associated with T_FH_ cells and sustained expression of naïve T cell genes associated with pT_CM_ cells (Fig. 3, b and f). Although initially divergent, the two branches converged upon effector states with more phenotypic similarity to each other than their respective developmental branches. Thus, while environmental signals may initially diversify effector populations, there remains the possibility of later plasticity.

To gain insights into the signaling pathways associated with these divergent cell fates, we analyzed the gene expression trends along the two differentiation pathways by specifying each branch endpoint and considering each trajectory separately (Fig. 3 g), generating a tool for dissecting the temporally ordered signaling pathways involved in the development of distinct T cell lineages. In addition to providing a description of relevant gene expression trends along the two branches below, we also created a publicly available resource allowing interrogation of temporal patterns of gene expression upon T cell activation to facilitate future studies of T cell lineage commitment available at https://cd4t-differentiation-dashboard.com.

The first branch consisted of 16 hpi cells that progressively upregulate TCR-dependent early activation genes, including *Cd69*, *Il2ra*, and NF-κB pathway-related genes (“TCR-hi”), and downregulate expression of naïve CD4^+^ T cell markers such as *Sell* and *Ccr7* (Fig. 3 g and Fig. S3 b). To determine the temporal dynamics of cytokine signaling pathways associated with T cell fate commitment, we first examined the expression of genes related to the IL-2 signaling pathway, due to its well-established role in regulating the choice between T_H_1 and T_FH_ fates by inhibiting Bcl6, the key transcription factor for T_FH_ development (Ballesteros-Tato et al., 2012). Analysis of IL-2 signaling pathway genes in the first branch demonstrated up-regulation of IL-2 expression downstream of TCR signaling which was sustained until entry into the cell cycle (Fig. 3 h). In contrast to a previous study describing mutually exclusive IL-2 and IL2Ra chain expression by activated CD4^+^ T cells in IL-2 reporter mice (DiToro et al., 2018), we observed concordant upregulation of *Il2ra* in IL-2–expressing cells (Fig. S3 c). Early T_H_1 branching cells retained *Il2ra* expression and demonstrated increased *Il2rb* (CD122) (Fig. 3 h). Furthermore, *Il2ra* was not expressed by early T_FH_ cells, implicating the dynamic regulation of the IL-2 receptor subunits as the primary tuner of IL-2 responsiveness and consequently of T_H_1 versus T_FH_ fate, which is in agreement with a previously described role for IL-6 mediated inhibition of IL2Rb expression in germinal center T_FH_ cells (Papillion et al., 2019). While Id3 expression was found to delineate T_H_1 cells with memory potential as early as day 7 after infection with LCMV-Armstrong (Shaw et al., 2022), its expression was not identified at the early time points after infection with *L. monocytogenes*, suggesting that acquisition of memory potential in T_H_1 phenotype cells may be a relatively rare event not captured in this dataset or may occur at later time points not analyzed here (https://cd4t-differentiation-dashboard.com).

The second branch was exclusively comprised of 40 hpi cells with significantly reduced TCR-dependent gene expression (“TCR-lo”), sustained *Ccr7*, and increasing memory cell gene expression, including *Tcf7* and *Cd27* (Hendriks et al., 2000; Zhou et al., 2010), suggesting that these represent the first pT_CM_ cells and arise directly from naïve T cells (Fig. 3 g and Fig. S3 b). Gene set enrichment analysis between the “TCR-lo” and “TCR-hi” differentiation trajectories confirmed differential expression of IL-2 signaling–related genes and revealed differential expression of type I IFN–related genes (Fig. S3 d), suggesting that effector and memory T cell fates result from naïve T cells that encounter antigen in distinct environmental niches. Analysis of cytokine related genes revealed distinct temporal patterns of IFN signaling between the two trajectories. Within cells along the “TCR-hi” trajectory, IFN stimulated genes (ISGs) were transiently upregulated prior to TCR activation genes, followed by upregulation of *Socs1* and termination of IFN signaling (Fig. 3 g and Fig. S3 b). In contrast, “TCR-lo” cells comprised of 40 hpi cells exhibited sustained ISG expression, and marked enrichment of *Ifnar1* and *Ifnar2* transcripts indicated that type I IFN response is temporally regulated, in part through IFN-α receptor expression (Fig. 3 i, Fig. S3 d, and Table S1). Analysis of cell surface IFNAR1 protein confirmed its increased expression in CD62L^+^ T_CM_ cells relative to their naïve and effector counterparts (Fig. S3 e). The emergence of the pT_CM_ differentiation pathway later in infection (40 hpi) in comparison with the earlier T_H_1 branch delineation (16 hpi) may reflect increased production of type I IFN by innate cells at this time point.

To experimentally validate the temporal correlation between cells that have received IFN signaling and cells that end up in the T_CM_ lineage, we generated dual reporter and fate-mapper mice by breeding the *Mx1*^*GFP*^ mice reporting on *Mx1* transcription from the endogenous locus (Uccellini and García-Sastre, 2018) with the transgenic *Mx1*^*Cre*^ mice (Kühn et al., 1995) and with mice harboring a *Rosa26*^*lox-STOP-lox-tdTomato*^ recombination reporter allele, in which tdTomato (tdT) expression irreversibly tags cells that have received a type I IFN signal. *Mx1*^*GFP*^ × tg*Mx1*^*Cre*^*Rosa26*^*lsl-tdT*^ mice were infected with L.m.-gp66 and assessed for history of IFN signaling (i.e., Mx1 expression) in antigen-specific CD4^+^ T cells identified using LLO:I-A^b^ or gp66:I-A^b^ tetramers on day 7 after infection (Fig. 3 j). It is noteworthy that the Mx1 transcript was not one of the top differentially expressed genes (DEGs) within the signature ISG gene set. Therefore, *Mx1*^*GFP*^ expression likely faithfully reports on cells experiencing the strongest type I IFN signal as noted in the original study (Uccellini and García-Sastre, 2018) rather than reflecting low tonic signaling or potential spurious expression of some ISGs in T cells. This analysis revealed an increased frequency of *Mx1* fate-mapped cells amongst CD62L^+^ pT_CM_ cells relative to their T_FH_ or T_H_1 counterparts (Fig. 3, k and l), confirming the predicted trajectory identified earlier in which cells receiving type I IFN signals adopt a pT_CM_ fate.

### Naïve T cells are transcriptionally heterogeneous

**Figure 4.**
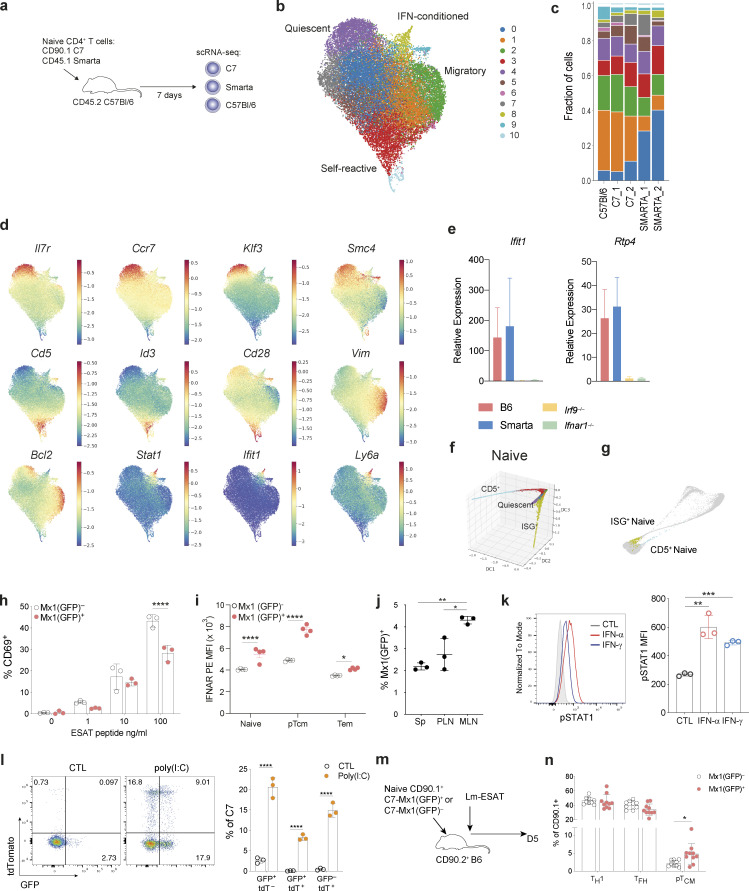
**Naïve CD4**^**+**^ **T cell heterogeneity. (a)** Experimental strategy for profiling splenic naïve CD4^+^ T cells. TCRγδ^−^PBS57/CD1d tetramer^−^NK1.1^−^TCRβ^+^CD4^+^CD25^−^CD44^lo^CD62L^hi^ cells, were sorted from the spleen of C7 or Smarta tgTCR mice and adoptively transferred into CD45.2 B6 recipients. After 7 days, naïve host B6 and tgTCR CD4^+^ T cells were isolated and profiled by scRNA-seq. **(b)** UMAP visualization of 28,146 naïve CD4^+^ T cells, colored by Phenograph cluster. **(c)** Fraction of cells within each naïve CD4^+^ T cell cluster detected across strains and biological replicate samples colored by Phenograph cluster as shown in Fig. 1 b. C57Bl/6, 3,026 cells; C7_1, 2,675 cells; C7_2, 6,159 cells; Smarta_1, 7,834 cells; Smarta_2, 8,452 cells. **(d)** UMAP colored by MAGIC imputed expression of cluster-defining genes. **(e)** Quantitative real-time PCR analysis of ISGs in bulk splenic naïve CD4^+^ T cells from B6, Smarta, *Irf9*^−/−^, or *Ifnar1*^−/−^ mice. **(f)** Naïve T cells, as shown in Fig. 4 b, visualized using diffusion map embedding of the first three DCs, with distinctive ISG^+^, quiescent, and CD5^+^ “self-reactive” cells labeled. Cells colored by Phenograph cluster as in Fig. 4 b. **(g)** Cells in the ISG^+^ naïve CD4^+^ T cell cluster (cluster 8, Fig. 4 b) and CD5^+^ naïve cells (cluster 10, Fig. 4 b), highlighted in distinct differentiation trajectories of early CD4^+^ T cell differentiation from Fig. 3 g. **(h)** Proportion of CD69^+^ cells among naïve C7 CD4^+^ T cells cultured for 36 h with irradiated T cell–depleted splenocytes (as antigen-presenting cells) and limiting concentrations of ESAT peptide. **(i)** IFNAR1 mean fluorescence intensity (MFI) on Mx1(GFP)^+^ versus Mx1(GFP)^−^ populations in uninfected mice. **(j)** Mx1(GFP)^+^ naïve CD4^+^ T cells are present in all lymphoid tissues, with varying frequencies across anatomically distinct LNs; PLN and MLN. **(k)** Representative MFI (histogram) (left) and summary bar graph (right) showing expression of pSTAT1 in sort-purified naïve Smarta CD4^+^ T cells treated in vitro with IFN-α or IFN-β for 4 h. **(l)** Naïve tdTomato^−^GFP^−^ CD4^+^ T cells from C7 × *Mx1*^*GFP*^ × tg*Mx1*^*Cre*^*Rosa26*^*lsl-tdT*^ mice were adoptively transferred into congenic B6 mice, administered the following day with poly(I:C) i.p. Representative flow cytometry plot showing expression of tdTomato and GFP in transferred splenic C7 naïve CD4^+^ T cells, 4 days after treatment (left) and quantification (right). **(m)** Mx1(GFP)^*+*^ or Mx1(GFP)^−^ naïve C7 CD4^+^ T cells were adoptively transferred into congenic B6 mice, subsequently infected intravenously with L.m.-ESAT and analyzed at 5 dpi. **(n)** Proportion of splenic pT_CM_ (CD62L^+^), T_H_1 (T-bet^+^CXCR5^−^), and T_FH_ (T-bet^−^CXCR5^+^) T cells amongst transferred C7 T cells at 5 dpi. Results are from one experiment representative of 4 (j), 3 (m and n), 2 (e, i, k, and l) independent experiments with *n* = 3 (e, j, and l), *n* = 4 (i), *n* = 9 (n) mice per group and three replicate wells in h and k. Statistical significance by two-way ANOVA (h, i, and l); one-way ANOVA (j and k); unpaired *t* test (n); *P < 0.05; **P < 0.01; ***P < 0.001; ****P < 0.0001. Error bars: means ± SEM.

**Figure S4.**
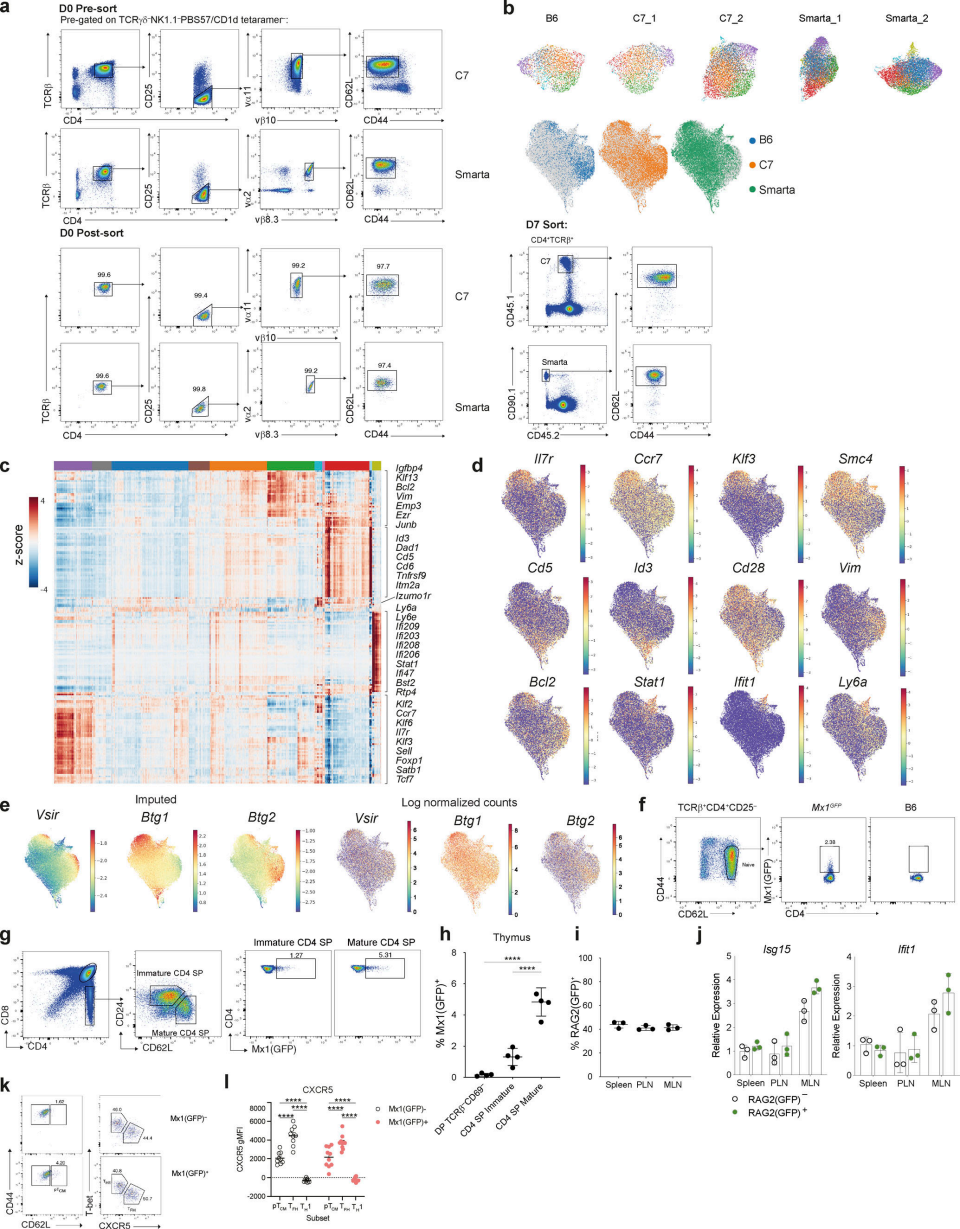
**Characterization of naïve CD4^+^ T cell heterogeneity.** Related to Fig. 4.** (a)** Representative flow cytometry showing sorting strategy for isolation of naïve CD4^+^ Smarta or C7 cells on day 0, prior to transfer into CD45.2 recipient mice (upper and middle panel). On day 7 after transfer, tgTCR T cells and host B6 naïve CD4^+^CD25^−^CD44^lo^CD62L^hi^ T cells were sorted for scRNA-seq analysis (right panel). At day 7, tgTCR T cells retained their naïve cell surface phenotype. **(b)** Top: UMAP visualization of individual naive CD4^+^ T cell replicate samples, each colored by their collective Phenograph clustering from Fig. 4 b performed after batch-correction. Bottom: UMAP colored by donor cell origin sorted according to a: B6 (blue) from one donor mouse, C7 (orange) and SMARTA (green) each from two donor mice. **(c)** Heatmap showing imputed expression of top 50 DEGs across splenic naïve T cell clusters (log_2_FC > 0.5, FDR < 0.01). The colored bar at the top of the heatmap shows the assignment of cells to clusters labeled in Fig. 4 b. Genes of interest are shown on the right. **(d)** Log-normalized expression values for comparison with imputed expression Fig. 4 d. **(e)** UMAP of naïve CD4^+^ T cells colored by imputed (left) or log normalized (right) expression of genes implicated in maintenance of naïve T cell quiescence. **(f)** Representative flow cytometry of naïve CD4^+^ T cells from the spleen (Sp) of *Mx1*^*GFP*^ mice. **(g)** Representative flow cytometry demonstrating gating strategy for analysis of CD4^+^ thymocyte populations from Mx1(GFP)^+^ mice. **(h)** Summary graph showing frequency of Mx1^+^ cells for each thymocyte population gated in g. Increased frequency of Mx1^+^ cells is observed as cells undergo progressive maturation from CD4^+^CD8^+^ DP thymocytes to mature single positive (SP) CD4^+^ T cells. Representative of two independent experiments, *n* = 4. Statistical significance was determined by one-way ANOVA; ****P < 0.0001. **(i)** Frequency of RAG2(GFP)^+^ cells within PLN, MLN, or spleen. **(j)** Expression of indicated ISGs in RAG2(GFP)^+^ or RAG2(GFP)^−^ naïve T cells, determined by qPCR. Representative of two independent experiments of *n* = 3. **(k)** Representative flow cytometric analysis of immune cell composition within the spleen of recipient mice, 5 days after infection, demonstrating frequency of pT_CM_ (CD62L^+^), T_H_1 (T-bet^+^CXCR5^−^), and T_FH_ (T-bet^−^CXCR5^+^) cells amongst transferred C7 T cells. **(l)** CXCR5 geometric MFI (gMFI) in T cell subsets from k. Representative of two independent experiments. Statistical significance determined by two-way ANOVA; ****P < 0.0001.

Our trajectory analysis identified two distinct branches of cells that emerge from naïve T cells. Given previous reports of naïve CD4^+^ T cell heterogeneity, we wondered whether some naïve T cells may already be poised for a particular cell fate upon activation (ElTanbouly et al., 2020). To address this, we profiled naïve (TCRγδ^−^PBS57/CD1d tetramer^−^NK1.1^−^TCRβ^+^CD4^+^CD25^−^CD44^lo^CD62L^hi^) mouse CD4^+^ T cells using scRNA-seq. To exclude inadvertent capture of recently activated T cells retaining a naïve phenotype, we also analyzed congenically marked naïve CD4^+^ T cells expressing two different transgenic (tg) TCRs in the absence of their cognate antigens. To exclude potential variation in the host environment, we adoptively transferred CD90.1^+^ C7 (Gallegos et al., 2008) and CD45.1^+^ Smarta (Oxenius et al., 1998) tgTCR CD4^+^ T cells into CD90.2^+^CD45.2^+^ C57Bl/6 (B6) recipients, and 7 days later isolated both transferred tgTCR and host naïve CD4^+^ T cells for scRNA-seq analysis (Fig. 4 a and Fig. S4 a). Phenograph clustering of 28,146 cells identified 11 clusters shared across the three strains (Fig. 4, b and c; and Fig. S4 b) (Levine et al., 2015). Differential gene expression analysis demonstrated cluster-specific signatures indicative of phenotypic and functional heterogeneity (Fig. 4 d; Fig. S4, c and d; and Table S2). Cluster 4 was distinguished by increased expression of genes known to regulate T cell quiescence, including transcription factors *Foxp1* (Feng et al., 2011) and *Klf2* (Kuo et al., 1997), the regulator of mRNA abundance *Btg1* (Hwang et al., 2020), and the chromatin condensin subunit *Smc4* (Rawlings et al., 2011) (Fig. 4 d and Fig. S4, c–e). Cells in clusters 3 and 10 exhibited heightened self-reactivity as evidenced by increased expression of CD5 and CD6, reflecting stronger tonic TCR signaling induced by self-peptide-MHCII complexes (Fig. S4 c) (Azzam et al., 1998). Cluster 2 cells expressed genes related to the cytoskeleton including *Vim*, *Ezr* (Villin2), *Actin1*, and *Emp3* suggesting their enhanced migratory properties (Fig. S4 c). Both cluster 2 and cluster 4 expressed *Vsir,* encoding the inhibitory cell-surface molecule VISTA, recently shown to regulate naïve T cell quiescence (Fig. S4 e) (ElTanbouly et al., 2020). Whilst the transcriptional features of cells in these clusters may be determined by intrinsic factors, a subset of naïve T cells (cluster 8) was distinguished by high levels of expression of ISGs, indicating that these cells were sensing either type I or II IFNs (Fig. 4 d; and Fig. S4, c and d). By quantitative PCR (qPCR), ISG expression was almost undetectable in bulk naïve CD4^+^ T cells isolated from either *Irf9*^−/−^ or *Ifnar1*^−/−^ mice (Prigge et al., 2015; Matsuyama et al., 1993) (Fig. 4 e), confirming that exogenous type I IFN, likely IFN-α or IFN-β, was responsible for inducing ISG expression in naïve T cells.

Diffusion map analysis of naïve T cells highlighted a continuous phenotypic spectrum between naïve CD4^+^ T cells with two branches emerging from the quiescent cell state. One branch expressed high levels of CD5, indicative of high tonic TCR signaling, and the second branch contained IFN-sensing cells (Fig. 4 f). Analysis of *Cd5* or ISG expression of C7 T cells undergoing differentiation during *L. monocytogenes* infection (Fig. 3 a) confirmed that these two cell states appeared at the junctions of the previously identified effector differentiation trajectories (Fig. 4 g). CD5^+^ naïve cells connected to the T_H_1 fate trajectory (Fig. 3 g), a finding consistent with a recent study identifying a role for tonic TCR signaling in negatively regulating early T_FH_ cell lineage commitment (Bartleson et al., 2020). Thus, naïve T cells with their stereotypical transcriptional phenotypes may have a predetermined “activation energy” that may poise cells to adopt different fates. To determine whether IFN signaling–experienced naïve T cells had altered differentiation potential in comparison to their unexperienced counterparts, we examined the corresponding cell subsets from C7 mice × *Mx1*^*GFP*^ harboring a reporter for the type I IFN inducible gene, *Mx1* (Fig. S4 f) (Uccellini and García-Sastre, 2018). We sorted Mx1(GFP)^+^ or Mx1(GFP)^−^ naïve C7 T cells and stimulated them in vitro with irradiated T cell–depleted splenocytes and varying doses of cognate antigen. At 36 h after activation, a reduced proportion of type I IFN–experienced cells had upregulated CD69 (Fig. 4 h), suggesting that prior type I IFN exposure is associated with diminished downstream TCR signaling.

A surprising feature of naïve T cells was the consistent but small percentage of Mx1(GFP)^+^ cells at steady state, suggesting that only a subset of naïve T cells is exposed to or responsive to type I IFN. This could reflect a spatially restricted niche for type IFN signaling or, alternatively, different thresholds of sensitivity to type I IFN signaling. In support of the latter, analysis of cell surface IFNAR1 expression in CD4^+^ T cells from *Mx1*^*GFP*^ mice demonstrated a spectrum with the highest levels observed amongst cells sensing type I IFN (Mx1[GFP]^+^) (Fig. 4 i), suggesting that varying expression of IFNAR1 may be a determinant of type I IFN sensitivity.

To explore the environmental versus developmental causes of naïve CD4^+^ T cell heterogeneity due to type I IFN exposure, we characterized naïve T cell responsiveness to type I IFN signaling across different tissues. In *Mx1*^*GFP*^ mice, naïve GFP^+^ CD4^+^ T cells were observed with varying frequencies across anatomically distinct LNs (Fig. 4 j). In the thymus, *Mx1*^*GFP*^ was upregulated during immature to mature CD4 SP thymocyte transition (Fig. S4, g and h). Given the presence of ISG(GFP)^+^ CD4^+^ thymocytes, one possibility was that the presence of ISG^+^ naive CD4^+^ T cells in the periphery represented recent thymic emigrants (RTEs). However, using a RAG2-GFP reporter to identify RTEs, we found that the proportion of RTEs amongst naïve CD4^+^ T cells was similar across peripheral lymphoid tissues (Fig. S4 i) and ISG expression was equivalent between RAG2(GFP)^+^ and RAG2(GFP)^−^ naïve T cells (Fig. S4 j). These findings suggested that IFN response gene activation in the thymus was transient and that IFN signaling in peripheral naïve CD4^+^ T cells is distinct from IFN signaling in the thymus.

To assess the responsiveness of naïve CD4^+^ T cells to type I IFN, we stimulated sorted, naïve Smarta T cells with recombinant IFN-α in vitro*.* pSTAT1 expression observed 4 h after treatment (Fig. 4 k) confirmed that naïve T cells can respond to type I IFN in a manner uncoupled from TCR activation. Whilst constitutive IFN-β expression has been detected in lymphoid tissues (Lienenklaus et al., 2009), type I IFNs are typically associated with inflammation and play a critical role in antiviral responses. To determine if the IFN response of naïve CD4^+^ T cells could be dynamically regulated in vivo, we employed *Mx1*^*GFP*^ × tg*Mx1*^*Cre*^*Rosa26*^*lsl-tdT*^ mice bred to the C7 tgTCR mice. We transferred naive GFP^−^tdT^−^ C7 CD4^+^ T cells into congenic mice that were subsequently treated with poly(I:C). Analysis of tdT expression 4 days after treatment demonstrated that up to 50% of naïve CD4^+^ T cells had upregulated ISGs by reporter expression of tdT and GFP (Fig. 4 l). Furthermore, a significant proportion of tdT^+^ cells lacked Mx1(GFP) expression, which is consistent with the transient nature of IFN signaling and suggests the naïve CD4^+^ T cell heterogeneity observed is not a fixed state of naïve T cells but may rather reflect environmental signals experienced by the naïve T cells transiently as they circulate within or throughout lymphoid tissues. In control mice, ∼5% of transferred cells expressed GFP or tdT, further confirming the homeostatic type I IFN response of peripheral naïve CD4^+^ T cells.

To establish the functional significance of type I IFN sensing in naïve T cells in vivo, we adoptively transferred Mx1(GFP)^+^ or Mx1(GFP)^−^ naïve C7 T cells and infected mice with L.m.-ESAT (Fig. 4 m). Analysis of transferred cells 5 days after infection demonstrated an increased frequency of pT_CM_ cells expressing CD62L and intermediate for CXCR5 expression amongst transferred Mx1(GFP)^+^ cells (Fig. 4 n; and Fig. S4, k and l). Mx1(GFP)^+^ cells had a similar propensity as Mx1(GFP)^−^ counterparts to differentiate into T_FH_ or T_H_1 cells (Fig. 4 n) and Mx1(GFP)^−^ cells also contributed to pT_CM_, suggesting while Mx1(GFP)^+^ may exhibit a lineage bias toward pT_CM_ they do not represent the exclusive precursor pool. Together, these findings suggest that IFN-signaling in naïve CD4^+^ T cells poises them for CD62L^+^ pT_CM_ differentiation and supports our finding that IFN-signaling is associated with the T_CM_ fate. While additional genetic tools enabling precise temporal interference with IFN signaling in the course of CD4^+^ T cell activation are needed for further understanding of pT_CM_ differentiation, our results suggest that heterogeneity in naïve T cells, induced by signals in the environment, may shape their fate prior to antigen stimulation. Collectively these data demonstrate that naïve T cells are responsive to environmental cues in the periphery, which impart transcriptional heterogeneity and alter their differentiation potential in response to infection.

### Type I IFN signaling in naïve CD4^+^ T cells in human disease

**Figure 5.**
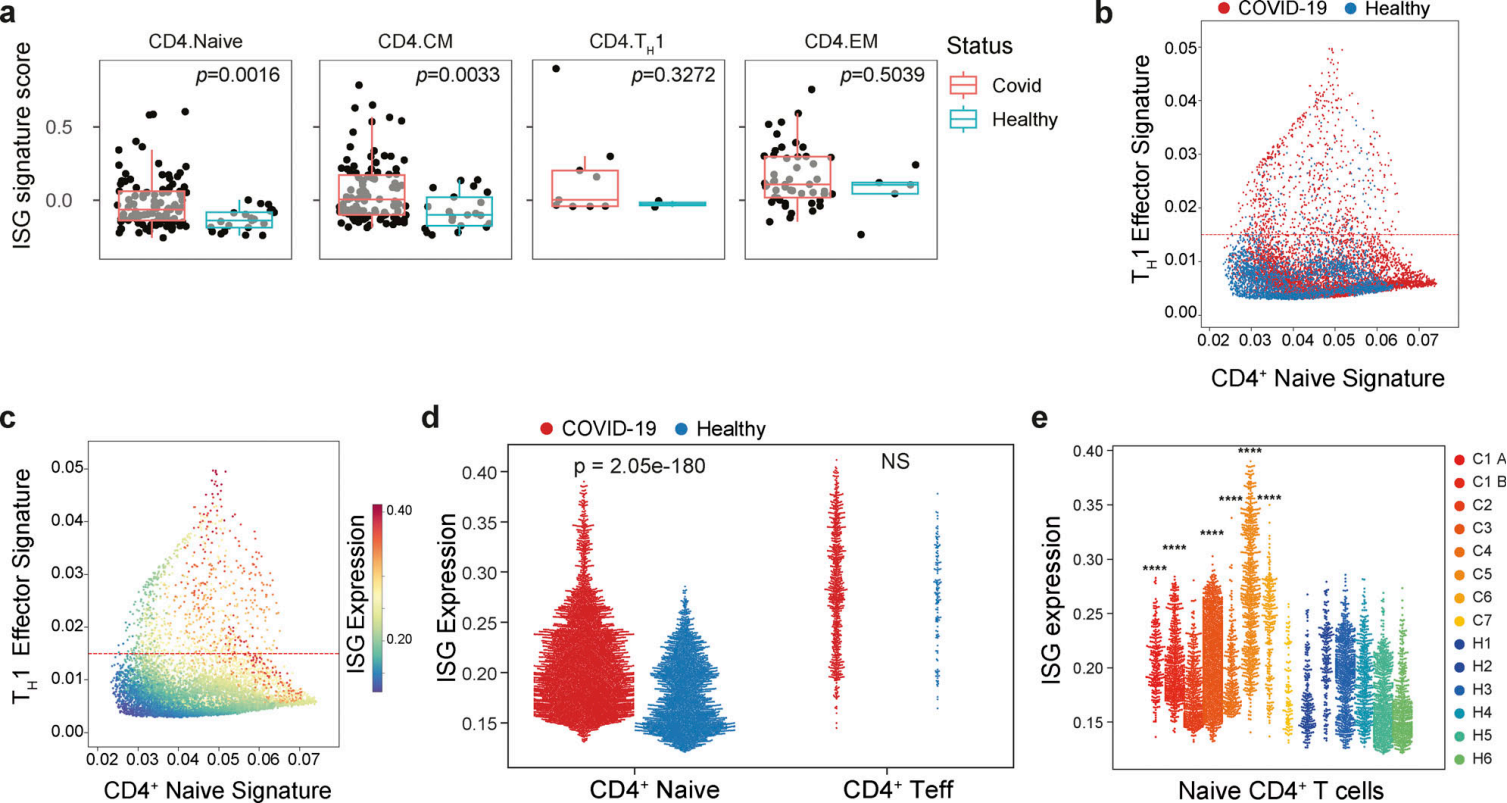
**Type I IFN signaling in naïve CD4^+^ T cells in COVID-19. (a)** ISG expression in patients with acute severe COVID-19 (Stephenson et al., 2021). ISG signature scores were averaged within each subset for each sample, with only samples having >10 cells for a particular CD4^+^ subset (central memory [CM], effector memory [EM], naïve, T_H_1) being used. Differential ISG signature score values between COVID-19 and healthy samples were assessed by a two-sided Wilcoxon rank-sum test. (COVID-19, *n* = 101; healthy control, *n* = 21). **(b–e)** ISG expression (as in Fig. 4 b) in patients with acute severe COVID-19 (Wilk et al., 2020). **(b and c)** Scatter plot of peripheral blood CD4^+^ T cell transcriptomes from healthy donors (H1–H6) or patients with acute severe COVID-19 (C1–C7). Each dot represents a cell, plotted by mean expression of the top 50 signature naïve T cell (x-axis) and T_H_1 effector genes, 6 days after viral infection (y-axis) and overlaid with disease status (b) or ISG signature expression (c). Cells above the dashed line representing the 10th percentile are considered effector T cells; cells below are considered naïve. **(d)** Naïve CD4^+^ T cell signature ISG genes (as in Fig. 4 b) are expressed higher amongst naïve CD4^+^ T cells, defined in Fig. 5 c, in patients with COVID-19 compared with healthy controls. **(e)** ISG signature gene expression in naïve CD4^+^ T cells for each individual patient and healthy control. Statistical significance by nonparametric Mann–Whitney *U* test between total COVID and total healthy populations (d), and between the individual COVID patient and total healthy populations (e); ****P < 0.0001.

Our finding that in vivo toll-like receptor (TLR) stimulation increases the number of ISG^+^ naïve T cells suggests that the pool of IFN-experienced naïve CD4^+^ T cells could be expanded by acute viral infection. To address the role of IFN-sensing in human T cells, we analyzed a large, well-annotated COVID-19 dataset generated by the Cambridge Institute of Therapeutic Immunology and Infectious Disease-National Institute for Health and Care Research (CITIID-NIHR) COVID-19 BioResource Collaboration (Stephenson et al., 2021) and found that donors who were infected with COVID-19 had higher expression of ISGs—as determined by applying a gene signature derived from the top 50 DEGs of the IFN-responsive cluster in Fig. 4 b—in circulating naïve CD4^+^ T cells, which are continuous with the pool of naïve CD4^+^ T cells transiting through secondary lymphoid organs (Mandl et al., 2012), and in central memory (CM) CD4^+^ T cells but not in effector memory (EM) CD4^+^ T cells or in T_H_1 cells (Fig. 5 a). To confirm these results, we turned to another publicly available scRNA-seq dataset of peripheral blood T cells from patients with acute COVID-19 infection (Wilk et al., 2020). Analysis of naïve T cells from this dataset revealed higher ISG expression in cells from infected patients compared with those from healthy controls (Fig. 5, b–e). In contrast, we did not observe differences in ISG expression between effector memory T cells from healthy versus infected patients suggesting that naïve CD4^+^ T cells are uniquely sensitive to fluctuations of this environmental cue. Together, these results demonstrate that in mice and humans, naïve CD4^+^ T cells can respond to type I IFN, and that the degree of response reflects their exposure in distinct niches, such as the intestinal draining LNs in mice, either in physiological settings (Fig. 4 j) or upon inflammatory perturbations that provoke type I IFN production (Fig. 4 l; and Fig. 5, a and d).

**Figure 6.**
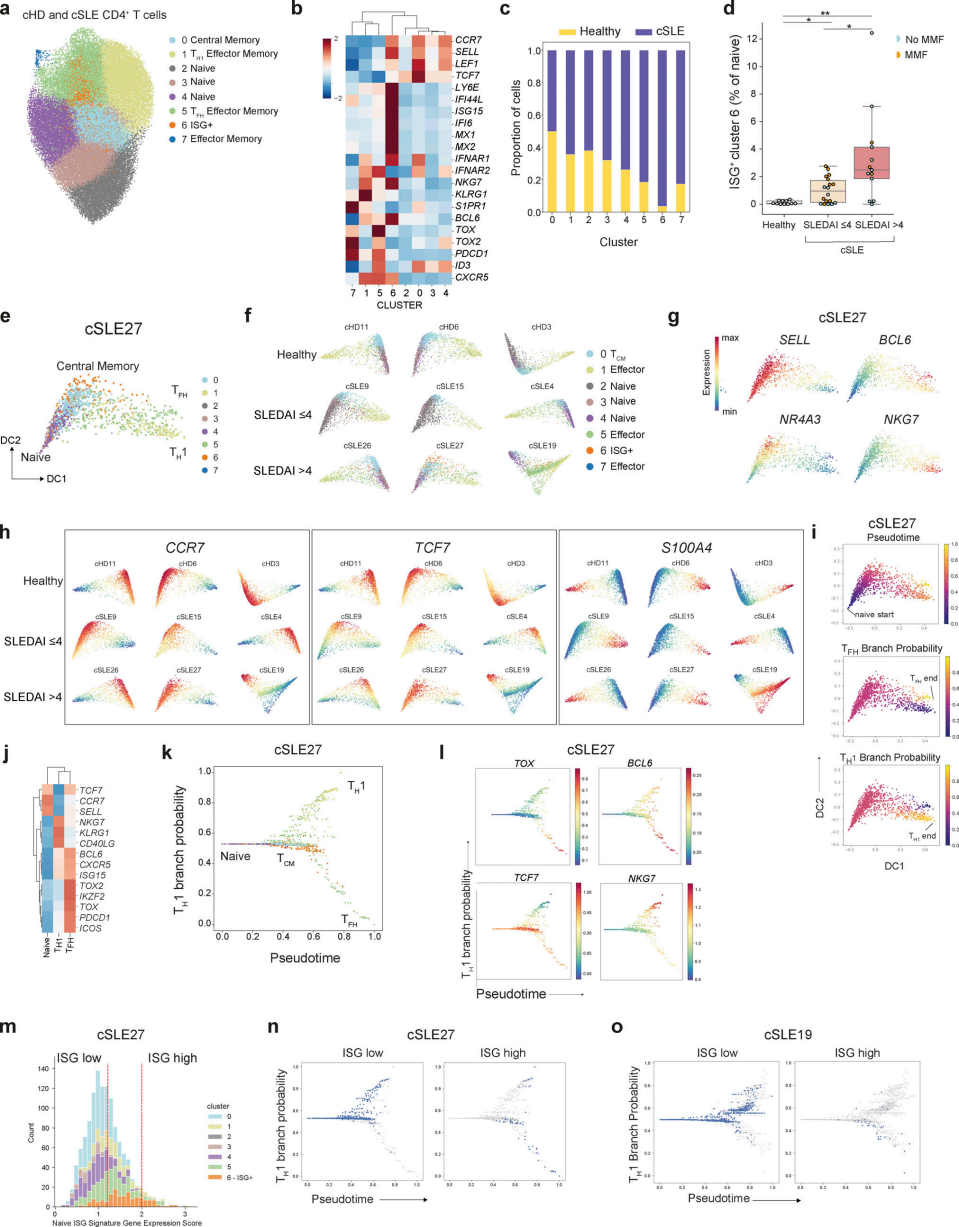
**Type I IFN signaling in naïve CD4^+^ T cells in SLE. (a)** UMAP visualization of 55,072 peripheral blood CD4^+^ T cells from 11 healthy children and 33 pediatric SLE patients. **(b)** Scaled expression of T cell lineage genes within clusters shown in a. **(c)** Proportion of healthy donor or cSLE derived cells in each cluster. **(d)** Proportion of cluster 6 ISG^+^ cells amongst naïve CD4^+^ T cells across healthy donors and cSLE patients, grouped according to low (SLEDAI ≤ 4) or high (SLEDAI > 4) disease activity. Two patients with an incomplete SLEDAI assessment were excluded from this analysis. **(e)** Diffusion map visualization of CD4^+^ T cells from an individual cSLE patient (cSLE_27) with high disease activity, colored by their cluster identity. **(f)** Individual diffusion maps from three representative healthy controls, three representative patients with low disease activity (SLEDAI ≤ 4) or three representative patients with high disease activity (SLEDAI > 4). **(g)** Diffusion map visualization of CD4^+^ T cells from an individual cSLE patient (cSLE_27) with high disease activity, colored by their expression of T cell lineage genes. **(h)** Individual diffusion maps (as in f) colored by imputed expression of indicated genes. **(i)** Palantir pseudotime and branch probabilities illustrating two differentiation trajectories from naïve to T_H_1 or T_FH_ cells. **(j)** Heatmap showing scaled expression of T cell lineage genes across the naïve and terminal effector memory cell states identified in e. **(k and l)** Reconstruction of effector T cell differentiation for patient cSLE27. T_H_1 branch probability across pseudotime, with cells (dots) colored by cluster identity (k), expression of T cell lineage genes (l). **(m)** Distribution of expression of mean ISG signature score for each cluster (as in Fig. 4 b) from patient cSLE_27. Cells below the 60th percentile were classified as “ISG-low,” and cells in the top 5% were labeled as “ISG-high.” **(n)** T_H_1 branch probability across pseudotime, with cells (dots) colored by level of ISG expression, for an individual patient, cSLE_27. ISG-low cells adopt a T_H_1 effector memory phenotype, whereas high levels of ISG expression are associated with a T_CM_ or T_FH_ memory phenotype. **(o)** T_H_1 branch probability across pseudotime, with cells (dots) colored by level of ISG expression, for an individual patient, cSLE_19. Statistical significance determined by Mann–Whitney test (d); *P < 0.05; **P < 0.01.

To study this possibility in a human disease setting other than infection, we considered that increased type I IFN production is a pathological feature of some autoimmune diseases, including SLE. A recent analysis of single-cell peripheral blood immune transcriptomes from a cohort of pediatric SLE patients (cSLE) revealed enhanced ISG expression in several cell types, including CD4^+^ T cells, compared with healthy controls (Nehar-Belaid et al., 2020). We reanalyzed this dataset to examine the effect of type I IFN signaling on naïve CD4^+^ T cell differentiation during autoimmune inflammation. Clustering of 55,072 CD4^+^ T cells from 33 cSLE patients and 11 pediatric healthy donors revealed the full spectrum from naïve to effector/memory CD4 T cell states represented in both SLE patients and in healthy controls (Fig. 6, a and b), including one ISG^hi^ naïve T cell cluster (cluster 6) that was almost exclusively comprised of cells from cSLE patients (Fig. 6 c). Similar to mouse ISG^+^ naïve CD4^+^ T cells, cluster 6 cells were distinguished from ISG^−^ naïve T cells by *LY6E* expression and had increased expression of *IFNAR1* (Fig. 6 b). As previously reported, patients with severe disease (SLEDAI > 4) displayed heightened proportions of ISG^+^ naïve CD4^+^ T cells (Fig. 6 d). Notably, unlike naïve cells, central memory cells were not overrepresented in the peripheral blood of SLE patients and healthy controls. This may be because T_CM_ cells are typically enriched in lymphoid tissues unlike their effector counterparts and their proportions in the peripheral circulation may not accurately reflect their numbers in the lymphoid tissues. We employed diffusion maps to visualize the relationship between ISG^+^ naïve T cells and their effector/memory counterparts for individual healthy donors or patients stratified by disease severity (Fig. 6, e and f). The first diffusion component (DC) separated naïve and effector memory T cells whilst the second component separated naïve and T_CM_ cells (Fig. 6 e), with increasing expression of *TCF7* and *NR4A3* (Fig. 6, g and h). This pattern was highly reproducible across individual donors irrespective of disease status (Fig. 6 h). To further assess the differentiation potential of ISG^+^ naïve CD4^+^ T cells, we deployed Palantir on cells for an individual patient with severe disease (cSLE_27). This analysis identified terminal T_H_1 and T_FH_ states based on canonical gene expression (Fig. 6, i and j).

Notably, ISG^+^ cells (cluster 6) follow the trajectory from naïve to T_CM_ cells expressing *TCF7* (Fig. 6, k and l) recapitulating our observations in the mouse *L. monocytogenes* infection model. To determine the fate of ISG^+^ cells, we first binned cells according to their level of ISG expression (Fig. 6 m) and then tracked their effector/memory fate along pseudotime (Fig. 6 n). Strikingly, ISG^lo^ cells were present almost exclusively within the T_H_1 effector memory branch, whilst cells with the highest levels of ISG expression exhibited features associated with T_CM_ and T_FH_ clusters at the bifurcation of the two branches and in the lower branch, respectively (Fig. 6 n). Analysis of differentiation trajectories for an additional patient with severe disease (cSLE_19) confirmed this finding (Fig. 6 o). Collectively, these studies suggest that naïve CD4^+^ T cell heterogeneity in mice and humans is dynamic in response to environmental type I IFN levels in viral infections and type I IFN–associated autoimmune diseases and suggest a role for type I IFN in regulating naïve T cells.

## Discussion

The ontogeny of pT_CM_ has remained elusive, with different models proposed to explain the emergence of pT_CM_ from either naïve or effector T cells. Our finding of pT_CM_ cells at the peak of the effector T cell response is in agreement with two recent studies reporting single-cell analyses of effector CD4^+^ T cell responses against LCMV (Andreatta et al., 2022; Shaw et al., 2022). The overlapping transcriptional phenotype between pT_CM_ and T_FH_ cells may account for difficulties resolving these distinct cell fates. By analyzing CD4^+^ T cells in their naïve state and at early time points after activation, we were able to identify the early emergence of pT_CM_ from naïve T cells. Our finding of early pT_CM_ differentiation is reminiscent of recent observations of precursors of central memory CD8^+^ T cells days before peak effector expansion (Kretschmer et al., 2020; Lin et al., 2016).

The observed enhancement of TCR signaling-dependent genes in early T_H_1 versus pT_CM_ cells is in overall agreement with previous studies reporting a role for increased TCR signal strength in driving T_H_1 commitment over T_FH_/pT_CM_ (Snook et al., 2018). However, our fate-mapping analysis of “naturally TCR barcoded” naïve CD4^+^ T cells argues strongly against an intrinsic role for the TCR itself as a dominant determinant of divergent T cell fates. Furthermore, distinguishing features of pT_CM_ and T_H_1 cells revealed by our analysis of early differentiation of naïve T cells expressing a transgene encoded TCR reflect potential modulation of TCR signaling by cell-extrinsic factors rather than intrinsic differences in TCR signaling strength conferred by TCR affinity for cognate antigen. The increased expression of type I IFN receptor and ISGs in cells with low levels of TCR signaling, coupled with the reduced expression of TCR-dependent molecules in IFN-sensing naïve T cells, suggest a potential role for type I IFN in the regulation of T cell fate through modulation of TCR signaling. The modulation of PI3K/Akt/Erk activity by IFN-α was notably reported to lead to altered expression of TCF1, a critical transcription factor for memory T cell differentiation (Li et al., 2020a; Gullicksrud et al., 2017), with diminished generation of stem cell-like memory cells in settings of genetic IFNAR1 deficiency or therapeutic IFNAR1 blockade (Li et al., 2020a).

By delineating the temporal order of gene expression, we uncovered two distinct pathways of naïve CD4^+^ T cell differentiation toward effector and memory cells in vivo. Our data suggest that the timing of activation plays a key role in determining the fate of naïve T cells. Naïve CD4^+^ T cells that undergo activation soon after infection, likely sense distinct cytokine environments given the temporally restricted IL-2 production by activated T cells, as well as the production of cytokines by antigen-presenting cells in response to stimulation via TLR and other innate immune receptors. Reduced TCR signaling in cells activated later in the infection course may reflect altered TCR signal strength due to likely shifts in the composition of antigen-presenting cell pool with varying cell surface expression of costimulatory and coinhibitory molecules and densities of cognate peptide-MHC complexes. Indeed, PD-1 signaling was recently shown to regulate early CD8^+^ memory differentiation (Johnnidis et al., 2021), suggesting that an encounter with PD-L1 expressing antigen-presenting cells may be a key determinant of memory formation.

In addition, our studies revealed heterogeneity amongst peripheral naïve CD4^+^ T cells and specifically identified naïve T cells poised to adopt specific cell fates upon activation. These results are consistent with recent studies describing heterogeneity amongst naïve CD4^+^ T cells in mice and humans (ElTanbouly et al., 2020; Gustafson et al., 2022, *Preprint*). An unexpected finding in our studies was that homeostatic cytokine sensing imparts naïve CD4^+^ T cell heterogeneity and impacts their fate decisions. While our study focused on type I IFN signaling, it is noteworthy that the environmental prevalence of type II cytokines was also recently found to influence naïve T cell responses (Even et al., 2024). This was associated with a diminished proportion of naïve ISG^+^ T cells with a concurrent reduction in overall proliferative potential, highlighting how environmental signal integration may be reflected in the naïve T cell pool. Although the functional significance of IFN-sensing of naïve CD4^+^ T cells remains to be established in CD8^+^ T cells, type I IFN sensitivity was shown to be coincident with the CD5 hi cells within the naïve CD8^+^ cell population and was attributed to self-reactivity, which resulted in expression of Ly6C (Ju et al., 2021). Interestingly, Ly6C^+^ CD8^+^ T cells preferentially differentiated into short-lived effector cells (Ju et al., 2021), whereas in our studies type I IFN exposure of naïve CD4^+^ T cells predisposed them toward a precursor central memory phenotype. While together these findings suggest that naïve T cell heterogeneity can be imparted by environmental cues, their effects on differentiation biases in CD8^+^ and CD4^+^ T cells appear discordant. Finally, a recent study identified a role for homeostatic IFN signaling in regulating baseline activation of a broad array of immune cells that determined vaccination responses in healthy individuals (Kotliarov et al., 2020), suggesting that IFN-signaling may alter the thresholds for immune cell activation or differentiation, consistent with previous reports identifying a role for IFN in licensing hematopoietic stem cell differentiation (Baldridge et al., 2010; Li et al., 2020b; Kim et al., 2016).

Thus, our studies suggest that the fate of a naïve T cell is impacted by environmental cues received before and during priming, independent of but combined with signals from the TCR. Understanding these signals may allow for modulation of the differentiation potential of naïve T cells through preconditioning regimes. These findings have implications for the design of adoptive T cell therapy in cancer and vaccination strategies, providing a potential therapeutic avenue for enhancing the memory potential of CD4^+^ T cells.

## Materials and methods

### Mice

C7, Smarta, *Irf9*^*−/−*^*, Ifnar1*^*−/−*^, tg*Mx1*^*Cre*^, and *Mx1*^*GFP*^ mice have been previously described (Gallegos et al., 2008; Oxenius et al., 1998; Matsuyama et al., 1993; Prigge et al., 2015; Uccellini and García-Sastre, 2018; Kühn et al., 1995). *R26*^*lsl-tdT*^ (Strain #:007914) and C57Bl/6 (CD45.2^+^) (Strain #:000664) mice were purchased from Jackson Laboratories. Mice were generated and treated under protocol 08-10-023 approved by the Sloan Kettering Institute (SKI) Institutional Animal Care and Use Committee. Specific pathogen–free mice were maintained in the SKI animal facility in accordance with institutional guidelines and ethical regulations. Germ-free C57Bl/6 mice were maintained in flexible isolators (Class Biologically Clean) at Weill Cornell Medicine. Animals were fed with autoclaved 5KA1 chow. Germ-free status was routinely checked by aerobic and anaerobic cultures of fecal samples for bacteria and fungi and by PCR of fecal DNA samples for bacterial 16S and fungal/yeast 18S genes. Both male and female mice were included in the study and we did not observe sex-dependent effects. All mice analyzed were age-matched (6–10 wk old). All animals used in this study had no previous history of experimentation and were naïve at the time of analysis.

### Cell isolation and flow cytometry

Lymphoid tissues were harvested, mashed through 100-μm strainers, washed with complete RPMI (cRPMI), and centrifuged. Spleen samples were treated with 1× ACK (155 mM ammonium chloride, 10 mM potassium bicarbonate, 100 nM EDTA pH 7.2) to lyse red blood cells and then washed with cRPMI and centrifuged. For cell sorting, in the analysis of adoptively transferred C7 CD4^+^ T cells in poly (I:C)-treated mice or L.m.-ESAT infected mice at early time points, cells were enriched using the Miltenyi CD4 Negative Selection Isolation Kit (Miltenyi) prior to cell sorting or analysis. For flow cytometric analysis, dead cells were excluded either by staining with LIVE/DEAD Fixable Violet, Ghost Dye Red 780, or Zombie NIR in PBS for 10 min at 4°C, prior to cell-surface staining. Cells were then incubated with anti-CD16/32 in staining buffer (2% FBS, 0.1% Na azide, in PBS) for 10 min at 4°C to block binding to Fc receptors. Surface staining for CXCR5 was performed at RT and staining with gp66:I-A^b^ tetramer was performed at 37°C for 45 min in cRPMI. All other extracellular antigens were stained for 20–30 min at 4°C in staining buffer. Intracellular phosphorylated STAT1 protein staining was performed with Phosflow Lyse/Fix Buffer, and Phosflow Perm Buffer III (BD Biosciences) according to the manufacturer’s protocol. Cells were washed with staining buffer before acquisition on a BD LSR II flow cytometer (Becton Dickinson) or Cytek Aurora. 123count eBeads were added to quantify absolute cell numbers. The antibodies used for flow cytometry and FACS are listed in Table S3.

### Real time qPCR

Naïve T cells were sorted directly into buffer RLT (Qiagen). Total RNA was extracted from cells using RNeasy Plus Micro kit (Qiagen) and reverse transcription was carried out with Superscript VILO IV master mix according to manufacturer instructions. qPCR reactions were set up in 384-well format in 10 µl using Power SYBR Green PCR Master Mix following manufacturer instructions. PCR was carried out on an Applied Biosystems 7900HT instrument using default settings. Expression of target genes was normalized to β-actin. Primer sequences are detailed in Table S4.

### *L. monocytogenes* infection and poly (I:C) treatment

L.m.-ESAT and L.m.-gp66 strains were provided by Marc Jenkins. For scRNA-seq of C7 effector T cell differentiation, 3 × 10^6^ sorted naïve CD4^+^CD25^−^CD44^lo^CD62L^hi^Vα11^+^Vβ10^+^ C7 T cells were adoptively transferred into congenic CD45.2^+^ C57Bl/6 mice. Mice were injected intravenously with 1 × 10^7^ colony-forming units (CFU) of L.m.-ESAT. In experiments comparing Mx1(GFP)^+^ versus Mx1(GFP)^−^ C7 naïve CD4^+^ T cells, CD4^+^ Vα11^+^Vβ10^+^CD25^−^CD44^lo^CD62L^hi^ Mx1(GFP)^+^ or Mx1(GFP)^−^ T cells were sorted from enriched CD4^+^ T cells, pooled from mesenteric LN (MLN), peripheral LN (PLN), and spleen of two to four mice. 2–4 × 10^4^ cells were transferred into CD45.2^+^ B6 recipient mice. The following day, mice were injected intravenously with 1 × 10^7^ CFU of L.m.-ESAT. For analysis of wild-type gp66^+^:I-A^b^-specific T cells, C57Bl/6 or tg*Mx1*^*cre*^*R26*^*lsl-tdT*^ mice were infected with 1 × 10^7^ CFU of L.m.-ESAT. Spleens were harvested 7 days after infection. For in vivo poly(I:C) treatment 10^6^ splenic tdTomato^−^GFP^−^ naïve CD4^+^ T cells sorted from C7 × *Mx1*^*GFP*^ tg*Mx1*^*Cre*^*ROSA*^*lsl-tdTomato*^ mice were transferred into congenic C57Bl/6 recipient mice. 12 h later, mice were injected intraperitoneally with 200 μg of poly(I:C) (Invitrogen).

### In vitro cell culture

Naïve CD4^+^ Vα11^+^Vβ10^++^CD25^−^CD44^lo^CD62L^hi^ C7 Mx1(GFP)^+^ or Mx1(GFP)^−^ T cells were sort purified after enrichment with a CD4^+^ T cell negative selection kit (Miltenyi Biotec). T cell–depleted splenocytes were prepared using biotinylated antibodies against CD4, followed by antibiotin microbeads (Miltenyi Biotec), and irradiation at 450 rad. Naïve CD4^+^ T cells were cultured for 36 h with irradiated splenocytes at a ratio of 1:1 and varying concentrations of ESAT6 peptide (InvivoGen). For assessment of cytokine production, cells were restimulated for 3 h at 37°C/5% CO_2_ in restimulation media (cRPMI 1640 with 5% FBS, 50 ng ml^−1^ PMA [Sigma-Aldrich], 500 ng ml^−1^ ionomycin [Sigma-Aldrich], 1 μg ml^−1^ brefeldin A [Sigma-Aldrich], and 2 μM monensin [Sigma-Aldrich]). For in vitro IFN treatment, 250,000 sorted naïve vα2^+^Vβ5^+^ Smarta CD4^+^ T cells were cultured at 37°C/5% CO_2_ for 4 h with 1,000 IU/ml of IFN-α4 (PBL Assay Science) or 25 ng/ml IFN-γ (Peprotech).

### scRNA-seq

7 days prior to analysis, naïve CD4^+^ T cells from tgTCR CD45.1^+^ Smarta or CD90.1^+^ C7 mice were adoptively transferred into C57Bl/6 recipients. Splenic CD4^+^ T cells were enriched with the CD4^+^ T cell negative isolation kit (Miltenyi). Two biological replicates, each representing a pool of two to three spleens, were processed for each tgTCR strain. Cells were incubated with anti-CD16/32 in sorting buffer (2% FBS in PBS) for 10 min at 4°C to block binding to Fc receptors. Extracellular antigens were stained for 30 min at 4°C in a sorting buffer. Cells were washed and resuspended in a sorting buffer with SYTOX blue (Invitrogen) for the exclusion of dead cells. Live, Lin(TCRγδ^−^PBS57/CD1d tetramer^−^NK1.1^−^)^−^TCRβ^+^CD4^+^CD25^−^CD44^lo^CD62L^hi^Vα11^+^Vβ10^+^ (C7) or Vα2^+^Vβ8.3^+^ (Smarta) T cells were then sort-purified using an Aria II cell sorter (BD Bioscience). Sorted cells were pelleted and resuspended in PBS. ∼5 × 10^6^ cells were transferred into a CD45.2^+^ B6 mouse. 7 days later, splenic CD4^+^ naïve T cells were enriched using the CD4 T cell negative isolation kit and stained with cell surface markers, as outlined above. Live CD90.1^+^CD4^+^TCRβ^+^ C7 or CD45.1^+^CD4^+^TCRβ^+^ T cells and host CD45.2^+^TCRγδ^−^PBS57/CD1d tetramer^−^NK1.1^−^TCRβ^+^CD4^+^CD25^−^CD44^lo^CD62L^hi^ naïve cells were sorted into cRPMI, pelleted and resuspended in RPMI-2% FBS. Two biological replicates, each representing a pool of two to three spleens, were processed for each tgTCR strain.

For scRNA-seq analysis of in vivo C7 differentiation, 3 × 10^6^ naïve CD4^+^CD25^−^CD44^lo^CD62L^hi^Vα11^+^Vβ10^+^ C7 T cells, sorted from a pool of two spleens, were adoptively transferred into congenic CD45.2^+^ C57Bl/6 mice. Mice were injected intravenously with 1 × 10^7^ CFU of L.m.-ESAT 18 h later. Spleens were harvested 16 h (two replicates) and 40 h after infection. CD4^+^ naïve T cells were enriched with the CD4 T cell negative isolation kit and stained with cell surface markers, as outlined above. Congenically marked CD90.1^+^ C7 cells were sorted into cRPMI before being pelleted and resuspended in RPMI-2% FBS. Each sample represents cells sorted from one recipient.

scRNA-seq of FACS-sorted cell suspensions was performed on the Chromium instrument (10X Genomics) following the user guide manual (CG00052 Rev E) and using Single Cell 3′ Reagent Kit (v2). Each sample, containing ∼8,000 cells at a final dilution of 66–70 cells/µl, was encapsulated and barcoded following the manual. Viability was 82–85% for samples containing naïve cells, and 80–99% for samples containing effector cells, as confirmed with 0.2% (wt/vol) Trypan Blue staining. The encapsulated cells were lysed, and following reverse transcription, the barcoded cDNA was purified with DynaBeads and amplified by 14 cycles of PCR: 98°C for 180 s, 12× (98°C for 15 s, 67 °C for 20 s, 72°C for 60 s), and 72°C for 60 s. 50 ng of PCR-amplified barcoded cDNA was fragmented with the reagents provided in the kit, purified with SPRI beads, and the resulting DNA library was ligated to the sequencing adapter followed by indexing PCR: 98°C for 45 s; 12 cycles of (98°C for 20 s, 54°C for 30 s, and 72°C for 20 s), and 72°C for 60 s. The final DNA library was double-size purified (0.6–0.8×) with SPRI beads and sequenced on Illumina Nova-Seq platform (R1–26 cycles, i7–8 cycles, R2–70 cycles or higher). Sequencing depth for naïve cell samples was between 65 and 85 million reads per sample (9,660 reads per cell), and for effector cells, 175–240 million reads per sample (57,150 reads per cell).

### scTCR-seq

Approximately 12,000 gp66:I-A^b^ tetramer-positive T cells were sorted from an L.m.-gp66 infected mouse, 7 days after intravenous infection. The TCR libraries were prepared following the Chromium Single Cell Immune Profiling Solution protocol. Sorted gp66:I-A^b^ tetramer-positive cells (>80% viability) were encapsulated at a final concentration of ∼120 cells/µl. After the reverse transcription step the barcoded-cDNA was released from droplets and purified with DynaBeads, followed by 14 PCR cycles (98°C for 45 s; [98°C for 20 s, 67°C for 30 s, 72°C for 1 min] × 14; 72°C for 1 min). The resulting cDNA library was used to construct single cell 5′ gene expression and TCR VDJ enriched libraries. The library was fragmented, double-size selected with SPRI beads (avg. size 450 bp), reamplified, and sequenced on Illumina NextSeq platform (R1–26 cycles, R2–98 cycles, i7–8 cycles) at a depth of ∼50,000 reads/cell.

To construct TCR libraries, 10 ng of barcoded material was amplified by a two-step nested PCR (10 cycles of PCR (98°C for 45 s; [98°C for 20 s, 67°C for 30 s, 72°C for 1 min] × 10; 72°C for 1 min) followed by an additional 10 cycles of PCR (98°C for 45 s; [98°C for 20 s, 67°C for 30 s, 72°C for 1 min] × 10; 72°C for 1 min) using DNA primers provided in the kit. VDJ region–enriched libraries with average size of 600 bp were sequenced on an Illumina HiSeq 2500 instrument (R1–150 cycles, R2–150 cycles, i7–8 cycles) to obtain ∼5,000 reads per cell. Replicate gp66:I-Ab tetramer^+^ samples represent independent samples, processed at separate time-points.

### scRNA-seq computational analysis

#### Preprocessing and quality control

To construct a count matrix, FASTQ files were processed using the Sequence Quality Control (SEQC) package (Azizi et al., 2018) with mm10 mouse genome reference and default parameters for the 10X platform. SEQC performs demultiplexing, read alignment, multimapping read resolution, as well as cell barcode and UMI correction to generate a (cell × gene) count matrix. The pipeline also performs initial cell filtering: true cells are distinguished from empty droplets based on the cumulative distribution of total molecule counts, and cells with a high fraction of mitochondrial molecules (>20%) or low library complexity (i.e., cells that express very few unique genes) are removed.

The combined count matrices from SEQC contained 11,403 cells by 12,044 genes from early effector time points in Fig. 3 and 28,146 cells by 11,190 genes across five naïve samples in Fig. 4. On average, SEQC filters removed ∼5% of cells for mitochondrial content and ∼2% of cells for library complexity in each sample. These default filters were designed to be permissive, and cells that express <500 molecules were further filtered to remove any remaining low-quality cell libraries.

Data from day 7 gp66:I-Ab^+^ scTCR-seq were individually processed with the CellRanger (v3.1.0) 5′ RNA-seq and V(D)J pipeline, aligned to mm10-3.0.0 for genomic libraries and vdj_GRCm38_alts_ensembl-3.1.0 for TCR libraries. The two day 7 scTCR-seq replicates together contained 5,681 cells by 11,299 genes.

For each dataset, the filtered count matrix combining all included samples was normalized for library size, multiplied by the median of the total molecule count across all cells for numerical stability, and log_2_-transformed with a pseudocount of 0.1 for downstream analysis. We retained genes with expression in >10 cells. Putative doublets were identified with Scrublet (Wolock et al., 2019), and any cluster with substantial doublet annotation was removed prior to downstream analysis (5.9% of naïve cells, 1% of early timepoint cells, 4.4% of D7 cells).

Basic metrics for each of the datasets are available in Table S5.

#### Clustering

We clustered each dataset by applying Phenograph to the principal component analysis (PCA)-reduced expression matrix and setting k to 30. This parameter choice ensures the capture of small discrete populations but tends to over-cluster regions of more continuous transcriptional variation, as observed between archetypal naïve phenotypes and between primary T effector states. However, the adjacency and continuity of these phenotypic regions can be corroborated by their relation in diffusion space (Figs. 1 e, 2 c, and 4 f).

#### Differential gene expression tests

Differential expression analysis was performed by applying MAST (v1.10.0) to library-size-normalized, log-transformed unimputed data using the number of genes expressed in each cell as a covariate (Finak et al., 2015). Cluster-specific gene expression was determined by comparing within-cluster expression to expression in all remaining clusters under consideration. Genes with false discovery rate (FDR) <1e-10 and fold change >1.4 were considered to exhibit differential expression.

#### Gene signatures

For a given set of genes, we calculated a gene signature score for each cell as the average imputed and z-scored expression level across genes in the input set.

We utilized published microarray data (GSE43863) for sorted naïve, memory, and effector CD4^+^ T cell populations generated during acute LCMV infection (D6) and memory recall (Hale et al., 2013). For each profile in this dataset, we derived a gene set and associated signature as the top 50 DEGs by fold change in a one-vs-rest comparison. For cell cycle signatures, we used published G1/S and G2/M cell cycle signatures (Tirosh et al., 2016). The IL2-STAT5 signaling signature was obtained from the MSigDB hallmark collection (Subramanian et al., 2005).

#### Antigen-specific gp66:I-A^b+^ T effector processing and analysis

**Figure S5.**
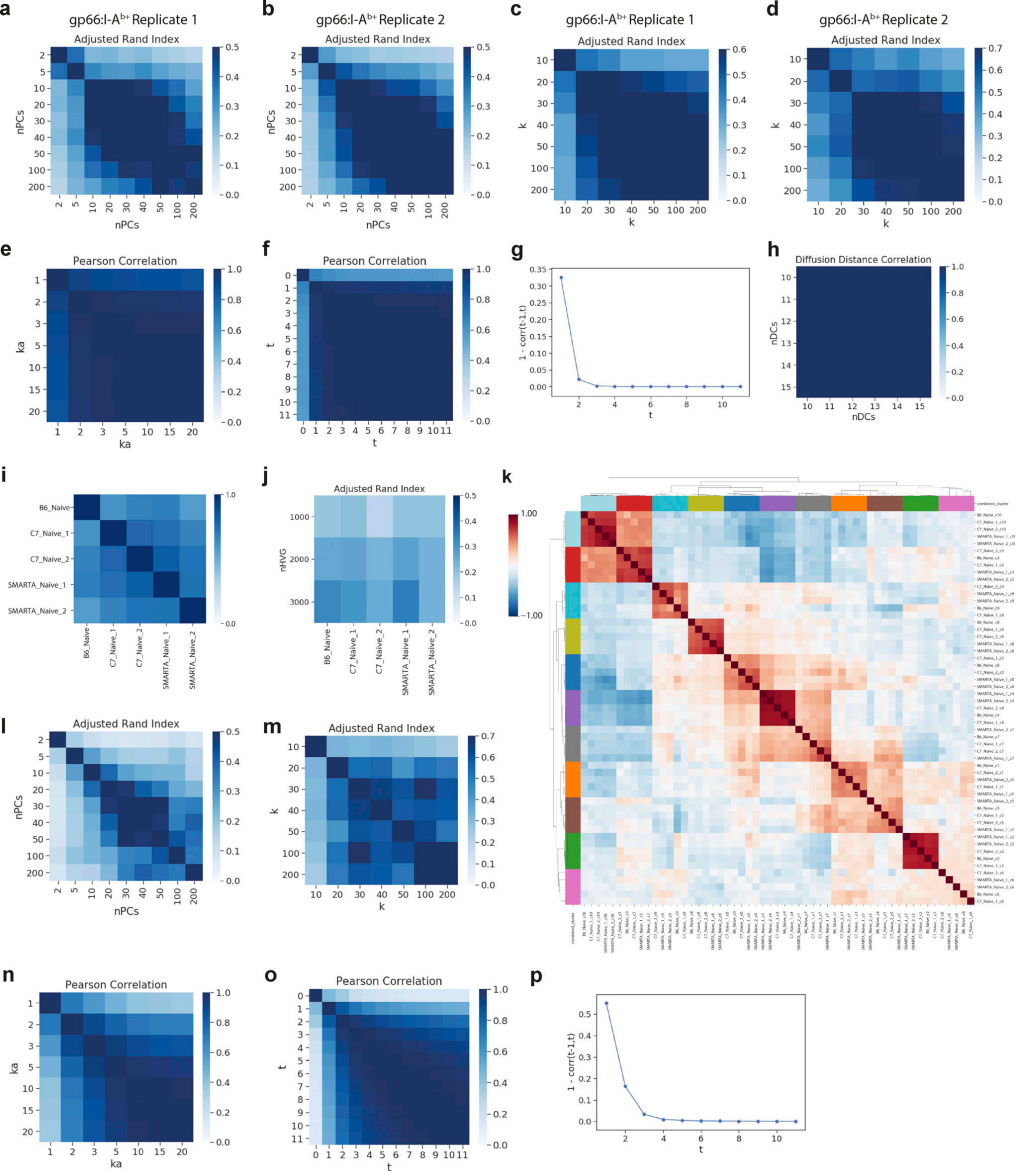
**Validation of parameters for scRNA-seq analysis of CD4^+^ T cells.** Related to Figs. 1, 2, 3, and 4. **(a–d)** Related to Figs. 1 and 2. Degree of overlap measured by adjusted Rand index between Phenograph clustering in gp66:I-A^b+^ CD4^+^ T cell replicates with varying input number of PCs (a and b) and k (c and d). **(e–h)** Related to Fig. 3. Validation of imputation and diffusion mapping for C7 differentiation during acute L.m.*-*ESAT infection. Average Pearson correlation per gene of MAGIC imputed expression values with varying input ka (e) and t parameters (f). **(g)** Average Pearson correlation distance per gene between successive applications of the MAGIC diffusion operator indicated by t. **(h)** Pearson correlation in cell–cell diffusion distances calculated for C7 differentiation dataset with varying numbers of diffusion components (DCs). **(i–k)** Related to Fig. 4. Validation of naïve CD4^+^ T cell batch-correction, clustering, and imputation. **(i)** Heatmap showing the overlap coefficient of DEGs recovered in each individual replicate sample after Phenograph clustering (20PCs, k = 30) followed by one-vs-rest cluster comparisons performed with MAST (log_2_FC > 0.5, FDR < 1e-10). **(j)** Heatmap showing the degree of overlap measured by the adjusted Rand index between clustering in individual samples using varying numbers of HVGs as compared with clustering in individual samples using all expressed genes. **(k)** Pearson correlation between average cluster expression profiles calculated separately in each individual sample. Colored bars at the top and sideshow the collective Phenograph clustering assignment of each profile. **(l–p)** Related to Fig. 4. Validation of clustering and imputation for naïve CD4^+^ T cell dataset. (**l and m)** Degree of overlap measured by adjusted Rand index between Phenograph clustering on the combined batch-corrected naïve T cell dataset with varying input number of PCs (l) and k (m). **(n and o)** Average Pearson correlation per gene of MAGIC imputed expression values with varying input ka (n) and t (o) parameters. **(p)** Average Pearson correlation distance per gene between successive applications of the MAGIC diffusion operator indicated by t.

We sampled two replicate 5′ scRNA-seq and scTCR-seq gp66:IA^b+^ Teff cell samples from a B6 mouse 7 days after infection (Figs. 1, 2, S1, and S2), and clustered and characterized each replicate separately for comparison. In each replicate, similar clustering, evaluated using the adjusted Rand index, was achieved for 20–100 input principal components (PCs), and input k ranging from 30 to 100 (Fig. S5, a–d). In one replicate, we removed a contaminating naïve cell cluster, likely resulting from non-specific tetramer binding, which was almost entirely composed of single-cell clones and exhibited high *Ccr7* and *Sell* expression. To assess the correspondence between gp66tet^+^ T cell replicates, we computed cluster centroids in each replicate dataset using the union of DEGs identified within each replicate separately. We then standardized (z-score) expression values for each gene across the cluster average profile within each dataset before calculating the Pearson correlation coefficient for each pairwise replicate-replicate cluster comparison (Fig. 2 f). To identify an inclusive set of DEGs distinguishing T_FH_1 and T_FH_2 subtypes, we took the union of DEGs found in T_FH_1-vs-rest and T_FH_2-vs-rest comparisons within each replicate (Fig. S1 c). To identify pT_CM_-specific genes, we merged clusters constituting the major pT_CM_, T_FH_, and T_H_1 differentiation states and computed DEGs in pT_CM_-vs-T_FH_ as well as pT_CM_-vs-T_H_1 comparisons (Fig. S1 f).

#### Antigen-specific gp66:I-A^b+^ T effector clonotype analysis

We used consensus CDR3 contigs defined by CellRanger to associate transcriptional profiles with distinct TCR clones and observed 76 and 47 clones with >5 cells in the scTCR-seq replicates. Each clonotype consists of multiple cells distributed across T cell phenotypic states, and we used an unsupervised strategy to define groups of clonotypes with similar phenotypic distributions. We based our approach on the MAGIC adaptive affinity matrix, employing the diffusion operator that determines cell phenotypic similarity, weighted by the major components of variation in the data. Each row of the adaptive affinity matrix is a unit-normalized vector that defines the local diffusion neighborhood of the corresponding cell. For each clonotype *c*, we defined a total neighborhood weight vector (*w*_*c*_) that aggregates the local neighborhoods of cells in the given clonotype, such that $w_{c}=\frac{1}{n_{c}}\underset{i\in c}{\sum }A_{i}$, where *A*_*i*_ is the *i*th row of the adaptive affinity matrix and *n*_*c*_ is the number of cells in clonotype *c*. The neighborhood weight vector captures regions of phenotypic similarity to a given clonotype on the data manifold. By associating clonotypes with broader phenotypic regions than their individual constituent cells, these neighborhood weight vectors can be used to evaluate the phenotypic similarity of distinct clonotypes. We clustered *w*_*c*_ vectors using Phenograph with a cosine distance metric and k = 10 to define clonotype groups occupying similar phenotypic spaces. These clonotype groups were associated with T_H_1-biased, T_FH_-biased, or mixed populations by inspecting their representation across gp66:I-A^b+^ T cell clusters. A ternary plot of the proportion of T_H_1, T_FH_, and pT_CM_ cells in each clonotype (Fig. S2 b) revealed that T_H_1-biased clonotypes contain >40% T_H_1 and <20% T_FH_ cells, while T_FH_-biased clonotypes contain >40% T_FH_ and <20% T_H_1 cells, corroborating our clonotype group annotations and emphasizing the dichotomy between T_FH_ and T_H_1 states found in clonotypes with phenotypic bias.

We also characterized patterns of phenotypic bias across clonotypes by counting the number of clonotypes with >5 cells observed in distinct combinations of pT_CM_, T_FH_, and T_H_1 phenotypes (Fig. 2 e). For this counting, we considered a clonotype to be represented in a given phenotype if one or more constituent cells were part of the given phenotypic cluster. To determine whether observed frequencies of phenotypic combinations (Fig. 2 e) diverged from expectation, we compared them to frequencies generated by randomly permuting clonotype labels. For each permutation, we shuffled labels across cell states without replacement, preserving the observed clonotype size distribution, and then counted clonotypes in each distinct phenotypic combination. We performed 500 permutations to obtain the background expectation in each phenotypic category and visualized deviations from this distribution by plotting observed–expected counts for each category as well as the randomized background dispersion (Fig. S2 c).

We further used GLIPH2 (Huang et al., 2020) to establish potential shared TCR specificity groups between gp66tet+ T cell replicate datasets. GLIPH2 aims to cluster TCR sequences that would bind the same MHC-restricted peptide antigen on the basis of both global TCR sequence similarity and the enrichment of short TCR sequence motifs relative to an unselected TCR reference set. We ran GLIPH2 using TCRβ chain consensus CDR3 contigs concatenated from both replicate datasets with all amino acids interchangeable and otherwise default parameters with the provided naïve mouse CD4 TCR set as reference. Of all significant TCRβ convergence groups, only two included >5 cell clones with identifiable phenotypic bias in both replicates.

#### C7 effector processing and analysis

To investigate the dynamics of early T effector induction, we sampled adoptively transferred splenic C7 TCR transgenic CD4 T cells 16 and 40 h after L.m.-ESAT infection (Fig. 3). To represent transcriptional heterogeneity prior to infection, we included a naïve C7 sample (with the largest number of cells) in our analysis. The samples displayed substantial shifts between time points, reflecting a rapidly changing environment and global phenotypic changes during early infection. We, therefore, utilized our data integration technique, Harmony (Nowotschin et al., 2019) (https://github.com/dpeerlab/Harmony), which is designed to connect time-adjacent scRNA-seq samples without strong integration assumptions. The algorithm identifies mutual nearest neighbors between adjacent time points, calculates a distance adjustment for these mNN edge weights, and incorporates them into an augmented affinity matrix suitable for downstream analysis. In the 40-h sample, we observed distinct clusters containing differentiated T cell phenotypes with high G1/S or G2/M scores (Fig. 3 c). Due to their highly proliferative signatures, these shared few nearest neighbors with other non-proliferative phenotypic states in either time point. To link initial non-proliferative steps of T cell activation with subsequent T cell maturation in proliferative states, we treated 40-h T cell clusters with high G1/S or G2/M scores as distinct and final time points. Ultimately, we obtained the complete augmented affinity matrix using Harmony by treating naïve, 16 h, non-proliferating 40 h, and proliferating 40 h T cells as time-adjacent input samples.

The augmented affinity matrix was subsequently used as input for visualization, clustering, diffusion maps, Palantir and MAGIC imputation. The combined early infection time course was visualized by ForceAtlas2 (https://github.com/bhargavchippada/forceatlas2) directly using the augmented affinity matrix as input (Jacomy et al., 2014). Imputed expression values calculated using the augmented affinity matrix are highly correlated (>0.95 Pearson coefficient) for input ka ranging from 3 to 20 and input t ranging from 2 to 11 (Fig. S5, e–g).

#### Palantir for early C7 effector differentiation during L.m.-ESAT

We used Palantir (Setty et al., 2019) to characterize pseudotime trajectories and gene trends toward different T cell activation fates in the early infection time-course dataset (Fig. 3 g). Palantir models cell fate as a continuous probabilistic process and calculates each cell’s position in pseudotime along with the probability that each cell reaches each terminal state, termed the branch probability. We ran Palantir using 10 DCs based on the eigengap, where the DCs were derived from the augmented affinity matrix produced with Harmony as described above. Diffusion distances were highly correlated (>0.95 Pearson coefficient) when calculated with a range of input DCs from 10 to 15 (Fig. S5 h). We set the starting cell in the naïve quiescent cluster and two terminal points in T_FH_- and T_H_1-biased states in the 40-h sample to capture the complete T cell activation and differentiation process (Fig. 3 d).

We observed two activation paths that both proceed toward T_H_ differentiation. To better resolve these divergent paths, we applied Palantir to a dataset in which we removed the common T_H_ differentiation stage by only retaining cells with pseudotime <0.7. This analysis identified two terminal states when a starting cell was specified in the naïve quiescent cluster; one pathway represented TCR-dependent activation and the other represented TCR-independent activation (Fig. 3 g and Fig. S3 b). We calculated gene expression trend along each pseudotime branch on MAGIC-imputed data using the generalized additive model available in Palantir, which is weighted by branch probabilities (Fig. 3 g and Fig. S3 b). We selected cells after branch divergence (pseudotime > 0.15) and performed differential expression between TCR-dependent (branch probability > 0.7) and TCR-independent cells to identify genes distinguishing the pathways.

#### Naive cell processing and analysis

To investigate naïve CD4^+^ T cell heterogeneity at steady state, we characterized sorted naïve splenic CD4 T cell populations from transgenic TCR (tgTCR) Smarta, C7, and clonally diverse B6 mice (Fig. 4). Initial clustering with Phenograph (20 PCs, k = 30) revealed a small contaminating B cell population that we removed before subsequent analysis. Batch effects caused naïve cell populations from each sample to form discrete populations when visualized together; however, when processed individually, we observed common determinants of heterogeneity in all naïve samples. For each individual naïve sample, we tabulated DEGs by clustering with Phenograph (20 PCs, k = 30), followed by one-vs-rest cluster comparisons with MAST (log_2_FC > 0.5, FDR < 1e-10). These gene sets were similar (>0.6 overlap coefficient) between naïve samples (Fig. S5 i).

To characterize shared axes of transcriptional variation in naïve populations, we selected the top 1,000 highly variable genes (HVGs) based on normalized dispersion within individual samples. We assessed the degree to which clustering is sensitive to the number of HVGs in individual samples and compared clustering based on HVGs with clustering based on all observed genes using the adjusted Rand index, finding good similarity between the solutions (Fig. S5 j). We then performed batch correction using mnnCorrect (k = 30 neighbors), with a Smarta replicate with the largest cell recovery and library size as the reference. For downstream analysis, we performed PCA on batch-corrected log-transformed HVG data. We selected 20 PCs (explaining ∼15% of the total variance) based on the rate of decay in explained variance per additional PC and retained the reduced matrix for downstream analysis including clustering, diffusion maps, and imputation. The combined batch-corrected naïve samples were visualized using UMAP (k = 30) (Fig. 4, b and d).

Visualization of the combined clustering on individually embedded samples illustrates that we retained naïve cell heterogeneity after batch correction (Fig. S4 b). To assess this quantitatively, we compared dimensionality reduction by PCA in individual samples with PCA in combined batch-corrected samples. We first projected individual sample data onto 20 sample-specific PCs and then reprojected it onto the 20 PCs from the total batch-corrected log data. When projected on the top PCs defined by the combined batch-corrected dataset, 60%, 54%, 65%, 69%, and 69% of the total explained variance from PCA in each naïve sample is retained. Furthermore, diffusion distances calculated within individual samples are well correlated (0.65, 0.64, 0.78, 0.86, and 0.93 Pearson coefficient) with diffusion distances calculated after batch correction. Finally, we assessed the similarity of cluster phenotypes across individual samples. Using the combined cluster labels produced using batch-corrected data, we computed cluster centroids in each individual sample separately on non-batch-corrected data. We then standardized (z-score) expression values for each gene across the cluster average profile within each individual sample before calculating the Pearson correlation coefficient for each pairwise sample and cluster comparison. Cluster phenotypes across all naïve samples are strongly self-similar, illustrating the phenotypic similarities shared in all naïve samples (Fig. S5 k).

Clustering on the combined naïve cells produced similar results for input PCs ranging from 20 to 50 and input k ranging from 30 to 200, evaluated using the adjusted Rand index (Fig. S5, l and m). Imputed expression for combined naïve cells is highly correlated (>0.95 Pearson coefficient) for input ka ranging from 5 to 20 and input t ranging from 3 to 11 (Fig. S5, n and o).

#### Diffusion maps and MAGIC imputation

To account for missing values in scRNA-seq, we employed MAGIC, a denoising method that imputes missing expression values based on data diffusion between cells with similar covariate gene relationships (van Dijk et al., 2018). We constructed the adaptive affinity matrix using k = 30, ka = 10, and t = 4 as input parameters, where t specifies the number of times the affinity matrix is powered for diffusion. As previously demonstrated (van Dijk et al., 2018), MAGIC imputed values are not very sensitive to input parameter choice, which we confirmed by varying these inputs (Fig. S5, e, f, and n–p). Following MAGIC, gene expression values were no longer sparse and followed better-structured distributions that align with the data manifold. MAGIC imputed data was used for visualization and gene signature calculation, but importantly, not for differential expression testing.

#### Human peripheral blood transcriptomes from COVID-19 patients

Processed count matrices related to the COVID-19 dataset generated by the CITIID-NIHR COVID-19 BioResource Collaboration (2021) study were downloaded from https://covid19cellatlas.org (Stephenson et al., 2021). An ISG expression signature was derived by taking DEGs from our MAST results for cluster 8 (IFN^+^ cluster) in the naïve samples. This list was filtered for one-to-one orthologs with humans, and the remaining 32 genes were used to compute average Z scores across all cells. To compare across samples, signature scores were averaged within each subset for each sample, with only samples having >10 cells for a particular CD4^+^ subset (CM, EM, Naïve, Th1) being used. Differential ISG signature score values between COVID-19 and healthy samples were assessed by a two-sided Wilcoxon rank-sum test (Fig. 5 a). We downloaded a preprocessed dataset containing PBMCs from five COVID-19 patients and six healthy controls (Wilk et al., 2020) from the CZ Biohub (Fig. 5 b). We isolated CD4^+^ T cell populations as annotated in the original publication and calculated gene signature scores for the “naïve Smarta” and “Day6 Ly6c^hi^ Cxcr5^−^” profiles as described above to partition naïve from potential effector cell states (Hale et al., 2013). We defined the set of potential naïve CD4^+^ T cells by exclusion of those with high effector (“Day6 Ly6c^hi^ Cxcr5^−^”) signature scores, using a conservative threshold that deviates substantially from majority background levels (∼10% of total CD4^+^ T cells). This threshold also retained cells with the highest naïve signature scores (Fig. 5, b and c). We additionally scored a signature derived from the top 50 DEGs of the IFN-responsive cluster in our naïve T cell characterization to demonstrate the presence of analogous IFN-responsive states in COVID patients. We tested for differences in IFN-responsive signature scores between COVID and healthy groups using the nonparametric Mann–Whitney *U* test between total COVID and total healthy populations, and between the individual COVID patient and total healthy populations (Fig. 5, d and e).

#### Human peripheral blood transcriptomes from SLE patients

A dataset containing all T cell populations from 33 SLE patients and 11 healthy control peripheral blood samples was generously provided by the authors of Nehar-Belaid et al. (2020), comprised of raw count data prefiltered for doublets and low library size transcriptomes (Fig. 6). After filtering genes for expression in at least 1,000 cells, this data contained 153,754 cells by 9,933 genes in total.

Given that we are interested in CD4^+^ T cell heterogeneity and differentiation, it was important to remove CD8^+^ T cell profiles before further analysis. Within each sample, we performed library-size normalization, scaled expression counts to 10,000 per cell, log_2_-transformed expression values with a pseudocount of 1, and z-scored expression for each gene. We developed a simple classification criterion from a small gene set (*Cd8b*, *Cd8a*, *Ccl5*, *Nkg7*, *Gzmm*, *Cd4*, *Cd40lg*, *Tnfrsf4*, *Itgb1*). These genes were selected for both their high expression and high correlation to CD4, CD8a, or CD8b. Within each sample, we took the first PC score using this gene set as input to define a composite score representing their expression. From all cells in a given sample, we selected cells with mutually exclusive expression of *Cd4* or *Cd8a/b* to serve as proxy labels for classification. We chose the classification threshold for the PC score that maximized Matthew’s correlation coefficient (>0.8 for 42/44 samples) and extended the classification criterion to the remaining cells in each sample. While this strategy cannot be guaranteed to remove all contaminating CD8 T cells, it did remove clusters composed exclusively of cells with *Cd8a/b* and little *Cd4* expression (probable CD8-specific cell states). If any CD8^+^ cells remained that could not be easily distinguished from CD4^+^ profiles, we reasoned that their presence would not substantially alter our inferences of CD4^+^ T cell behavior.

After filtering CD8 cells (average 64 ± 11% of total T cells per sample), raw count data from 55,748 CD4^+^ T cell profiles were compiled and expression values were collectively library-size normalized, scaled to 10,000 counts per cell, log_2_-transformed with a pseudocount of 1, and z-scored in each gene. Two small clusters of contaminating myeloid cells and neutrophils were removed prior to further analysis. With the retained CD4^+^ T cells (average 1,251 ± 683 cells per patient), we performed batch correction and integration using Scanorama with 50 PCs. Phenograph clustering and UMAP embedding were calculated using the resulting batch-corrected dimensionality reduced space and 30 nearest neighbors. A single cluster was defined by high expression of IFN-responsive elements (Fig. 6 a, ISG^+^ cluster). We tested for differences in the proportion of CD4^+^ T cells constituted by the ISG^+^ cluster across disease activity categories defined in Nehar-Belaid et al. (2020) using the nonparametric Mann–Whitney *U* test (Fig. 6 d).

We employed diffusion maps to examine the role of IFN response in ISG^+^ naïve T cells and their effector/memory counterparts for individual healthy donors or patients stratified by disease severity (Fig. 6, e and f). For this analysis, we chose samples with high cell number and phenotypic coverage as well as IFN-responsive gene expression and computed DCs independently for each sample. The DCs illustrate shared phenotypic variation and consistency across the analysis (Fig. 6 f). The first DC separates naïve and effector memory T cells whilst the second component separates naïve and T_CM_ cells (Fig. 6 e), with increasing expression of *TCF7* and *NR4A3* (Fig. 6, g and h). This pattern was highly reproducible across individual donors irrespective of disease status (Fig. 6 h). Notably, ISG^+^ cells follow the trajectory from naïve to T_CM_ cells (Fig. 6, e, f, n, and o), supporting our earlier finding that IFN-experienced naïve T cells are poised for T_CM_ fate. To further assess the differentiation potential of ISG^+^ naïve CD4^+^ T cells, we deployed Palantir on cells for an individual patient with severe disease (cSLE_27). We defined the potential phenotypic trajectory from naïve to T_H_1 and T_FH_-biased effector endpoints using Palantir, selecting the number of input DCs based on eigenvalue decay. For each DC, we included the cells with the minimum or maximum DC value as candidate start or endpoints for Palantir input and used the expression of canonical naïve, T_H_1, and T_FH_ genes to identify starting naïve and terminal T_H_1 and T_FH_ states (Fig. 6, i and j). To determine the fate of IFN-conditioned cells, we binned cells by ISG expression (Fig. 6 m) and then tracked their fate along pseudotime (Fig. 6, k–n). Strikingly, ISG^lo^ cells were present almost exclusively within the T_H_1 effector memory branch, whilst cells with the highest ISG expression exhibited increased T_CM_ and T_FH_ memory potential (Fig. 6, k–n). Analysis of differentiation trajectories for an additional patient with severe disease (cSLE_19) confirmed this finding (Fig. 6 o).

### Online supplemental material

Fig. S1 shows additional gene expression, analyses, and non-imputed UMAPs pertaining to Fig. 1. Fig. S2 shows additional analyses related to scTCR-seq of gp66:I-A^b+^ CD4^+^ T cells. Fig. S3 depicts individual UMAPs of different time points emphasized in Fig. 3 and additionally shows pseudotime gene expression trends and MsigDB Hallmark pathways enriched in the two differentiation trajectories. Fig. S4 shows the sorting strategy, post-sort purity, and replicate UMAPs of naïve CD4^+^ T cell data as well as analysis of IFN-inducible genes in RTEs. Fig. S5 depicts tested parameters and quality control for computational analyses performed on datasets shown in Figs. 1, 3, and 4. Table S1 lists the top marker genes for TCR-independent versus TCR-dependent differentiation trajectories as shown in Fig. 3. Table S2 lists the top marker genes for each naïve cell cluster shown in Fig. 4 b. Table S3 contains information for antibodies used in this study. Table S4 lists primer sequences for qPCR data shown in Fig. 4 and Fig. S4. Table S5 contains basic summary metrics for scRNA-seq datasets generated in this study.

## Supplementary Material

Table S1 contains top marker genes for TCR-independent versus TCR-dependent differentiation trajectories.

Table S2 contains top marker genes for each naïve cell cluster shown in Fig. 4 b.

Table S3 contains the list of antibodies used in this study.

Table S4 shows primer sequences for qPCR.

Table S5 contains basic summary metrics for scRNA-seq datasets generated in this study.

## Data Availability

The mouse sequencing data related to Figs. 1, 2, 3, and 4 are available through the Gene Expression Omnibus under accession GSE171527. The data related to Fig. 3 is available as a data browser at https://cd4t-differentiation-dashboard.com. All computational analyses on human data were performed on published datasets. The sequencing date for pediatric SLE patients has been published previously and deposited in the dbGAP database under accession number phs002048.v1.p1. COVID-19 datasets are available at https://covid19cellatlas.org and https://www.covid19cellatlas.org.

## References

[bib1] Andreatta, M., A. Tjitropranoto, Z. Sherman, M.C. Kelly, T. Ciucci, and S.J. Carmona. 2022. A CD4^+^ T cell reference map delineates subtype-specific adaptation during acute and chronic viral infections. Elife. 11:e76339. 10.7554/eLife.76339 35829695 PMC9323004

[bib2] Azizi, E., A.J. Carr, G. Plitas, A.E. Cornish, C. Konopacki, S. Prabhakaran, J. Nainys, K. Wu, V. Kiseliovas, M. Setty, . 2018. Single-cell map of diverse immune phenotypes in the breast tumor microenvironment. Cell. 174:1293–1308.e36. 10.1016/j.cell.2018.05.060 29961579 PMC6348010

[bib3] Azzam, H.S., A. Grinberg, K. Lui, H. Shen, E.W. Shores, and P.E. Love. 1998. CD5 expression is developmentally regulated by T cell receptor (TCR) signals and TCR avidity. J. Exp. Med. 188:2301–2311. 10.1084/jem.188.12.2301 9858516 PMC2212429

[bib4] Baldridge, M.T., K.Y. King, N.C. Boles, D.C. Weksberg, and M.A. Goodell. 2010. Quiescent haematopoietic stem cells are activated by IFN-gamma in response to chronic infection. Nature. 465:793–797. 10.1038/nature09135 20535209 PMC2935898

[bib5] Ballesteros-Tato, A., B. León, B.A. Graf, A. Moquin, P.S. Adams, F.E. Lund, and T.D. Randall. 2012. Interleukin-2 inhibits germinal center formation by limiting T follicular helper cell differentiation. Immunity. 36:847–856. 10.1016/j.immuni.2012.02.012 22464171 PMC3361521

[bib6] Bartleson, J.M., A.A. Viehmann Milam, D.L. Donermeyer, S. Horvath, Y. Xia, T. Egawa, and P.M. Allen. 2020. Strength of tonic T cell receptor signaling instructs T follicular helper cell-fate decisions. Nat. Immunol. 21:1384–1396. 10.1038/s41590-020-0781-7 32989327 PMC7578106

[bib7] van Dijk, D., R. Sharma, J. Nainys, K. Yim, P. Kathail, A.J. Carr, C. Burdziak, K.R. Moon, C.L. Chaffer, D. Pattabiraman, . 2018. Recovering gene interactions from single-cell data using data diffusion. Cell. 174:716–729.e27. 10.1016/j.cell.2018.05.061 29961576 PMC6771278

[bib8] DiToro, D., C.J. Winstead, D. Pham, S. Witte, R. Andargachew, J.R. Singer, C.G. Wilson, C.L. Zindl, R.J. Luther, D.J. Silberger, . 2018. Differential IL-2 expression defines developmental fates of follicular versus nonfollicular helper T cells. Science. 361:eaao2933. 10.1126/science.aao2933 30213884 PMC6501592

[bib9] ElTanbouly, M.A., Y. Zhao, E. Nowak, J. Li, E. Schaafsma, I. Le Mercier, S. Ceeraz, J.L. Lines, C. Peng, C. Carriere, . 2020. VISTA is a checkpoint regulator for naïve T cell quiescence and peripheral tolerance. Science. 367:eaay0524. 10.1126/science.aay0524 31949051 PMC7391053

[bib10] Esterházy, D., M.C.C. Canesso, L. Mesin, P.A. Muller, T.B.R. de Castro, A. Lockhart, M. ElJalby, A.M.C. Faria, and D. Mucida. 2019. Compartmentalized gut lymph node drainage dictates adaptive immune responses. Nature. 569:126–130. 10.1038/s41586-019-1125-3 30988509 PMC6587593

[bib61] Even, Z., A.P. Meli, A. Tyagi, A. Vidyarthi, N. Briggs, D.A. de Kouchkovsky, Y. Kong, Y. Wang, D.A. Waizman, T.A. Rice, . 2024. The amalgam of naive CD4^+^ T cell transcriptional states is reconfigured by helminth infection to dampen the amplitude of the immune response. Immunity. 57:1893–1907.e6. 10.1016/j.immuni.2024.07.006 39096910 PMC11421571

[bib11] Feng, X., H. Wang, H. Takata, T.J. Day, J. Willen, and H. Hu. 2011. Transcription factor Foxp1 exerts essential cell-intrinsic regulation of the quiescence of naive T cells. Nat. Immunol. 12:544–550. 10.1038/ni.2034 21532575 PMC3631322

[bib12] Finak, G., A. McDavid, M. Yajima, J. Deng, V. Gersuk, A.K. Shalek, C.K. Slichter, H.W. Miller, M.J. McElrath, M. Prlic, . 2015. MAST: A flexible statistical framework for assessing transcriptional changes and characterizing heterogeneity in single-cell RNA sequencing data. Genome Biol. 16:278. 10.1186/s13059-015-0844-5 26653891 PMC4676162

[bib13] Gallegos, A.M., E.G. Pamer, and M.S. Glickman. 2008. Delayed protection by ESAT-6-specific effector CD4^+^ T cells after airborne M. tuberculosis infection. J. Exp. Med. 205:2359–2368. 10.1084/jem.20080353 18779346 PMC2556792

[bib14] Glanville, J., H. Huang, A. Nau, O. Hatton, L.E. Wagar, F. Rubelt, X. Ji, A. Han, S.M. Krams, C. Pettus, . 2017. Identifying specificity groups in the T cell receptor repertoire. Nature. 547:94–98. 10.1038/nature22976 28636589 PMC5794212

[bib15] Gullicksrud, J.A., F. Li, S. Xing, Z. Zeng, W. Peng, V.P. Badovinac, J.T. Harty, and H.-H. Xue. 2017. Differential requirements for tcf1 long isoforms in CD8^+^ and CD4^+^ T cell responses to acute viral infection. J. Immunol. 199:911–919. 10.4049/jimmunol.1700595 28652395 PMC5531591

[bib16] Gustafson, C.E., Z. Thomson, Z. He, E. Swanson, K. Henderson, M.-P. Pebworth, L.Y. Okada, A.T. Heubeck, C.R. Roll, V. Hernandez, . 2022. Distinct heterogeneity in the naive T cell compartments of children and adults. bioRxiv. 10.1101/2022.10.04.510869 (Preprint posted October 07, 2022).

[bib17] Hale, J.S., B. Youngblood, D.R. Latner, A.U.R. Mohammed, L. Ye, R.S. Akondy, T. Wu, S.S. Iyer, and R. Ahmed. 2013. Distinct memory CD4^+^ T cells with commitment to T follicular helper- and T helper 1-cell lineages are generated after acute viral infection. Immunity. 38:805–817. 10.1016/j.immuni.2013.02.020 23583644 PMC3741679

[bib18] Hendriks, J., L.A. Gravestein, K. Tesselaar, R.A. van Lier, T.N. Schumacher, and J. Borst. 2000. CD27 is required for generation and long-term maintenance of T cell immunity. Nat. Immunol. 1:433–440. 10.1038/80877 11062504

[bib19] Huang, H., C. Wang, F. Rubelt, T.J. Scriba, and M.M. Davis. 2020. Analyzing the Mycobacterium tuberculosis immune response by T-cell receptor clustering with GLIPH2 and genome-wide antigen screening. Nat. Biotechnol. 38:1194–1202. 10.1038/s41587-020-0505-4 32341563 PMC7541396

[bib20] Hwang, S.S., J. Lim, Z. Yu, P. Kong, E. Sefik, H. Xu, C.C.D. Harman, L.K. Kim, G.R. Lee, H.-B. Li, and R.A. Flavell. 2020. mRNA destabilization by BTG1 and BTG2 maintains T cell quiescence. Science. 367:1255–1260. 10.1126/science.aax0194 32165587

[bib21] Jacomy, M., T. Venturini, S. Heymann, and M. Bastian. 2014. ForceAtlas2, a continuous graph layout algorithm for handy network visualization designed for the Gephi software. PLoS One. 9:e98679. 10.1371/journal.pone.0098679 24914678 PMC4051631

[bib22] Johnnidis, J.B., Y. Muroyama, S.F. Ngiow, Z. Chen, S. Manne, Z. Cai, S. Song, J.M. Platt, J.M. Schenkel, M. Abdel-Hakeem, . 2021. Inhibitory signaling sustains a distinct early memory CD8^+^ T cell precursor that is resistant to DNA damage. Sci. Immunol. 6:eabe3702. 10.1126/sciimmunol.abe3702 33452106 PMC8258400

[bib23] Ju, Y.-J., S.-W. Lee, Y.-C. Kye, G.-W. Lee, H.-O. Kim, C.-H. Yun, and J.-H. Cho. 2021. Self-reactivity controls functional diversity of naive CD8^+^ T cells by co-opting tonic type I interferon. Nat. Commun. 12:6059. 10.1038/s41467-021-26351-3 34663827 PMC8523551

[bib24] Khatun, A., M.Y. Kasmani, R. Zander, D.M. Schauder, J.P. Snook, J. Shen, X. Wu, R. Burns, Y.-G. Chen, C.-W. Lin, . 2021. Single-cell lineage mapping of a diverse virus-specific naive CD4 T cell repertoire. J. Exp. Med. 218:e20200650. 10.1084/jem.20200650 33201171 PMC7676493

[bib25] Kim, P.G., M.C. Canver, C. Rhee, S.J. Ross, J.V. Harriss, H.-C. Tu, S.H. Orkin, H.O. Tucker, and G.Q. Daley. 2016. Interferon-α signaling promotes embryonic HSC maturation. Blood. 128:204–216. 10.1182/blood-2016-01-689281 27095787 PMC4946201

[bib26] Kotliarov, Y., R. Sparks, A.J. Martins, M.P. Mulè, Y. Lu, M. Goswami, L. Kardava, R. Banchereau, V. Pascual, A. Biancotto, . 2020. Broad immune activation underlies shared set point signatures for vaccine responsiveness in healthy individuals and disease activity in patients with lupus. Nat. Med. 26:618–629. 10.1038/s41591-020-0769-8 32094927 PMC8392163

[bib27] Kretschmer, L., M. Flossdorf, J. Mir, Y.-L. Cho, M. Plambeck, I. Treise, A. Toska, S. Heinzel, M. Schiemann, D.H. Busch, and V.R. Buchholz. 2020. Differential expansion of T central memory precursor and effector subsets is regulated by division speed. Nat. Commun. 11:113. 10.1038/s41467-019-13788-w 31913278 PMC6949285

[bib28] Kühn, R., F. Schwenk, M. Aguet, and K. Rajewsky. 1995. Inducible gene targeting in mice. Science. 269:1427–1429. 10.1126/science.7660125 7660125

[bib29] Kuo, C.T., M.L. Veselits, and J.M. Leiden. 1997. LKLF: A transcriptional regulator of single-positive T cell quiescence and survival. Science. 277:1986–1990. 10.1126/science.277.5334.1986 9302292

[bib30] Levine, J.H., E.F. Simonds, S.C. Bendall, K.L. Davis, A.D. Amir, M.D. Tadmor, O. Litvin, H.G. Fienberg, A. Jager, E.R. Zunder, . 2015. Data-driven phenotypic dissection of AML reveals progenitor-like cells that correlate with prognosis. Cell. 162:184–197. 10.1016/j.cell.2015.05.047 26095251 PMC4508757

[bib31] Lienenklaus, S., M. Cornitescu, N. Zietara, M. Łyszkiewicz, N. Gekara, J. Jabłónska, F. Edenhofer, K. Rajewsky, D. Bruder, M. Hafner, . 2009. Novel reporter mouse reveals constitutive and inflammatory expression of IFN-beta in vivo. J. Immunol. 183:3229–3236. 10.4049/jimmunol.0804277 19667093

[bib32] Lin, W.W., S.A. Nish, B. Yen, Y.-H. Chen, W.C. Adams, R. Kratchmarov, N.J. Rothman, A. Bhandoola, H.-H. Xue, and S.L. Reiner. 2016. CD8^+^ T lymphocyte self-renewal during effector cell determination. Cell Rep. 17:1773–1782. 10.1016/j.celrep.2016.10.032 27829149 PMC5108530

[bib33] Li, W., L. Lu, J. Lu, X. Wang, C. Yang, J. Jin, L. Wu, X. Hong, F. Li, D. Cao, . 2020a. cGAS-STING-mediated DNA sensing maintains CD8^+^ T cell stemness and promotes antitumor T cell therapy. Sci. Transl. Med. 12:eaay9013. 10.1126/scitranslmed.aay9013 32581136

[bib34] Li, Y., W. Kong, W. Yang, R.M. Patel, E.B. Casey, T. Okeyo-Owuor, J.M. White, S.N. Porter, S.A. Morris, and J.A. Magee. 2020b. Single-cell analysis of neonatal HSC ontogeny reveals gradual and uncoordinated transcriptional reprogramming that begins before birth. Cell Stem Cell. 27:732–747.e7. 10.1016/j.stem.2020.08.001 32822583 PMC7655695

[bib35] Mandl, J.N., R. Liou, F. Klauschen, N. Vrisekoop, J.P. Monteiro, A.J. Yates, A.Y. Huang, and R.N. Germain. 2012. Quantification of lymph node transit times reveals differences in antigen surveillance strategies of naive CD4^+^ and CD8^+^ T cells. Proc. Natl. Acad. Sci. USA. 109:18036–18041. 10.1073/pnas.1211717109 23071319 PMC3497782

[bib36] Mandl, J.N., J.P. Monteiro, N. Vrisekoop, and R.N. Germain. 2013. T cell-positive selection uses self-ligand binding strength to optimize repertoire recognition of foreign antigens. Immunity. 38:263–274. 10.1016/j.immuni.2012.09.011 23290521 PMC3785078

[bib37] Matsuyama, T., T. Kimura, M. Kitagawa, K. Pfeffer, T. Kawakami, N. Watanabe, T.M. Kündig, R. Amakawa, K. Kishihara, A. Wakeham, . 1993. Targeted disruption of IRF-1 or IRF-2 results in abnormal type I IFN gene induction and aberrant lymphocyte development. Cell. 75:83–97. 10.1016/S0092-8674(05)80086-8 8402903

[bib39] Merkenschlager, J., S. Finkin, V. Ramos, J. Kraft, M. Cipolla, C.R. Nowosad, H. Hartweger, W. Zhang, P.D.B. Olinares, A. Gazumyan, . 2021. Dynamic regulation of T_FH_ selection during the germinal centre reaction. Nature. 591:458–463. 10.1038/s41586-021-03187-x 33536617 PMC7979475

[bib40] Nehar-Belaid, D., S. Hong, R. Marches, G. Chen, M. Bolisetty, J. Baisch, L. Walters, M. Punaro, R.J. Rossi, C.-H. Chung, . 2020. Mapping systemic lupus erythematosus heterogeneity at the single-cell level. Nat. Immunol. 21:1094–1106. 10.1038/s41590-020-0743-0 32747814 PMC7442743

[bib41] Nowotschin, S., M. Setty, Y.-Y. Kuo, V. Liu, V. Garg, R. Sharma, C.S. Simon, N. Saiz, R. Gardner, S.C. Boutet, . 2019. The emergent landscape of the mouse gut endoderm at single-cell resolution. Nature. 569:361–367. 10.1038/s41586-019-1127-1 30959515 PMC6724221

[bib42] Oxenius, A., M.F. Bachmann, R.M. Zinkernagel, and H. Hengartner. 1998. Virus-specific MHC-class II-restricted TCR-transgenic mice: Effects on humoral and cellular immune responses after viral infection. Eur. J. Immunol. 28:390–400. 10.1002/(SICI)1521-4141(199801)28:01<390::AID-IMMU390>3.0.CO;2-O 9485218

[bib43] Papillion, A., M.D. Powell, D.A. Chisolm, H. Bachus, M.J. Fuller, A.S. Weinmann, A. Villarino, J.J. O’Shea, B. León, K.J. Oestreich, and A. Ballesteros-Tato. 2019. Inhibition of IL-2 responsiveness by IL-6 is required for the generation of GC-T_FH_ cells. Sci. Immunol. 4:eaaw7636. 10.1126/sciimmunol.aaw7636 31519812 PMC6820141

[bib44] Pepper, M., A.J. Pagán, B.Z. Igyártó, J.J. Taylor, and M.K. Jenkins. 2011. Opposing signals from the Bcl6 transcription factor and the interleukin-2 receptor generate T helper 1 central and effector memory cells. Immunity. 35:583–595. 10.1016/j.immuni.2011.09.009 22018468 PMC3208313

[bib45] Persaud, S.P., C.R. Parker, W.-L. Lo, K.S. Weber, and P.M. Allen. 2014. Intrinsic CD4^+^ T cell sensitivity and response to a pathogen are set and sustained by avidity for thymic and peripheral complexes of self peptide and MHC. Nat. Immunol. 15:266–274. 10.1038/ni.2822 24487322 PMC3944141

[bib46] Prigge, J.R., T.R. Hoyt, E. Dobrinen, M.R. Capecchi, E.E. Schmidt, and N. Meissner. 2015. Type I IFNs act upon hematopoietic progenitors to protect and maintain hematopoiesis during pneumocystis lung infection in mice. J. Immunol. 195:5347–5357. 10.4049/jimmunol.1501553 26519535 PMC4655130

[bib47] Rawlings, J.S., M. Gatzka, P.G. Thomas, and J.N. Ihle. 2011. Chromatin condensation via the condensin II complex is required for peripheral T-cell quiescence. EMBO J. 30:263–276. 10.1038/emboj.2010.314 21169989 PMC3025460

[bib48] Setty, M., V. Kiseliovas, J. Levine, A. Gayoso, L. Mazutis, and D. Pe’er. 2019. Characterization of cell fate probabilities in single-cell data with Palantir. Nat. Biotechnol. 37:451–460. 10.1038/s41587-019-0068-4 30899105 PMC7549125

[bib49] Shaw, L.A., T.Z. Deng, K.D. Omilusik, K.K. Takehara, Q.P. Nguyen, and A.W. Goldrath. 2022. Id3 expression identifies CD4^+^ memory Th1 cells. Proc. Natl. Acad. Sci. USA. 119:e2204254119. 10.1073/pnas.2204254119 35858332 PMC9303986

[bib50] Skon, C.N., J.-Y. Lee, K.G. Anderson, D. Masopust, K.A. Hogquist, and S.C. Jameson. 2013. Transcriptional downregulation of S1pr1 is required for the establishment of resident memory CD8^+^ T cells. Nat. Immunol. 14:1285–1293. 10.1038/ni.2745 24162775 PMC3844557

[bib51] Snook, J.P., C. Kim, and M.A. Williams. 2018. TCR signal strength controls the differentiation of CD4^+^ effector and memory T cells. Sci. Immunol. 3:eaas9103. 10.1126/sciimmunol.aas9103 30030369 PMC6126666

[bib52] Stephenson, E., G. Reynolds, R.A. Botting, F.J. Calero-Nieto, M.D. Morgan, Z.K. Tuong, K. Bach, W. Sungnak, K.B. Worlock, M. Yoshida, . 2021. Single-cell multi-omics analysis of the immune response in COVID-19. Nat. Med. 27:904–916. 10.1038/s41591-021-01329-2 33879890 PMC8121667

[bib53] Subramanian, A., P. Tamayo, V.K. Mootha, S. Mukherjee, B.L. Ebert, M.A. Gillette, A. Paulovich, S.L. Pomeroy, T.R. Golub, E.S. Lander, and J.P. Mesirov. 2005. Gene set enrichment analysis: A knowledge-based approach for interpreting genome-wide expression profiles. Proc. Natl. Acad. Sci. USA. 102:15545–15550. 10.1073/pnas.0506580102 16199517 PMC1239896

[bib54] Tirosh, I., B. Izar, S.M. Prakadan, M.H. Wadsworth II, D. Treacy, J.J. Trombetta, A. Rotem, C. Rodman, C. Lian, G. Murphy, . 2016. Dissecting the multicellular ecosystem of metastatic melanoma by single-cell RNA-seq. Science. 352:189–196. 10.1126/science.aad0501 27124452 PMC4944528

[bib55] Tubo, N.J., A.J. Pagán, J.J. Taylor, R.W. Nelson, J.L. Linehan, J.M. Ertelt, E.S. Huseby, S.S. Way, and M.K. Jenkins. 2013. Single naive CD4^+^ T cells from a diverse repertoire produce different effector cell types during infection. Cell. 153:785–796. 10.1016/j.cell.2013.04.007 23663778 PMC3766899

[bib56] Uccellini, M.B., and A. García-Sastre. 2018. ISRE-reporter mouse reveals high basal and induced type I IFN responses in inflammatory monocytes. Cell Rep. 25:2784–2796.e3. 10.1016/j.celrep.2018.11.030 30517866 PMC6317368

[bib57] Weber, K.S., Q.-J. Li, S.P. Persaud, J.D. Campbell, M.M. Davis, and P.M. Allen. 2012. Distinct CD4^+^ helper T cells involved in primary and secondary responses to infection. Proc. Natl. Acad. Sci. USA. 109:9511–9516. 10.1073/pnas.1202408109 22645349 PMC3386110

[bib58] Wilk, A.J., A. Rustagi, N.Q. Zhao, J. Roque, G.J. Martínez-Colón, J.L. McKechnie, G.T. Ivison, T. Ranganath, R. Vergara, T. Hollis, . 2020. A single-cell atlas of the peripheral immune response in patients with severe COVID-19. Nat. Med. 26:1070–1076. 10.1038/s41591-020-0944-y 32514174 PMC7382903

[bib59] Wolock, S.L., R. Lopez, and A.M. Klein. 2019. Scrublet: Computational identification of cell doublets in single-cell transcriptomic data. Cell Syst. 8:281–291.e9. 10.1016/j.cels.2018.11.005 30954476 PMC6625319

[bib60] Zhou, X., S. Yu, D.-M. Zhao, J.T. Harty, V.P. Badovinac, and H.-H. Xue. 2010. Differentiation and persistence of memory CD8(+) T cells depend on T cell factor 1. Immunity. 33:229–240. 10.1016/j.immuni.2010.08.002 20727791 PMC2928475

