# Correction: Precursor central memory versus effector cell fate and naïve CD4^+^ T cell heterogeneity

**DOI:** 10.1084/jem.2023119310182024c

**Published:** 2024-10-30

**Authors:** Deeksha Deep, Herman Gudjonson, Chrysothemis C. Brown, Samuel A. Rose, Roshan Sharma, Yoselin A. Paucar Iza, Seunghee Hong, Saskia Hemmers, Michail Schizas, Zhong-Min Wang, Yuezhou Chen, Duane R. Wesemann, Virginia Pascual, Dana Pe’er, Alexander Y. Rudensky

Vol. 221, No. 11 | https://doi.org/10.1084/jem.20231193 | September 25, 2024

The authors regret that, throughout the article, the *Mx1*^*GFP*^ strain was accidentally labeled as transgenic (tg). Instead, it is the *Mx1*^*Cre*^ strain that is transgenic. The *Mx1*^*GFP*^ strain is a reporter from the endogenous locus, and the *Mx1*^*Cre*^ strain is a transgene. Therefore, tg*Mx1*^*GFP*^ has been changed to *Mx1*^*GFP*^ and *Mx1*^*Cre*^ has been changed to tg*Mx1*^*Cre*^ throughout the article, including within Fig. 3 j. In the Results, the sixth paragraph of the “A pT_CM_ differentiation pathway ... ” section has been reworded, with the changes shown in bold below. This correction does not change the original conclusions of the article. The errors appear in print and in PDFs downloaded before October 15, 2024.

## Results

**A pT_CM_ differentiation pathway arising from naïve CD4^+^ T cells** *(sixth paragraph)*

To experimentally validate the temporal correlation between cells that have received IFN signaling and cells that end up in the TCM lineage, we generated dual reporter and fate-mapper mice by breeding the ***Mx1*^*GFP*^ mice reporting on *Mx1* transcription from the endogenous locus** (Uccellini and García-Sastre, 2018) **with the transgenic *Mx1*^*Cre*^ mice** (Kühn et al., 1995) and with mice harboring a *Rosa26*^*lox-STOP-lox-tdTomato*^ recombination reporter allele, in which tdTomato (tdT) expression irreversibly tags cells that have received a type I IFN signal. ***Mx1*^*GFP*^ × tg*Mx1*^*Cre*^***Rosa26*^*lsl-tdT*^ mice were infected with L.m.-gp66 and assessed for history of IFN signaling (i.e., Mx1 expression) in antigen-specific CD4^+^ T cells identified using LLO:I-A^b^ or gp66:I-A^b^ tetramers on day 7 after infection (Fig. 3 j). It is noteworthy that the Mx1 transcript was not one of the top differentially expressed genes (DEGs) within the signature ISG gene set. Therefore, *Mx1*^*GFP*^ expression likely faithfully reports on cells experiencing the strongest type I IFN signal as noted in the original study (Uccellini and García-Sastre, 2018) rather than reflecting low tonic signaling or potential spurious expression of some ISGs in T cells. This analysis revealed an increased frequency of *Mx1* fate-mapped cells amongst CD62L^+^ pT_CM_ cells relative to their T_F_H or T_H_1 counterparts (Fig. 3, k and l), confirming the predicted trajectory identified earlier in which cells receiving type I IFN signals adopt a pT_CM_ fate.

